# Strongly Coupled Ag/Sn–SnO_2_ Nanosheets Toward CO_2_ Electroreduction to Pure HCOOH Solutions at Ampere-Level Current

**DOI:** 10.1007/s40820-023-01264-6

**Published:** 2023-12-13

**Authors:** Min Zhang, Aihui Cao, Yucui Xiang, Chaogang Ban, Guang Han, Junjie Ding, Li-Yong Gan, Xiaoyuan Zhou

**Affiliations:** 1 College of Physics and Center of Quantum Materials and Devices, Chongqing University, Chongqing, 401331 People’s Republic of China; 2 State Key Laboratory of Structural Chemistry, Fujian Institute of Research on the Structure of Matter, Chinese Academy of Sciences (CAS), Fuzhou, 350002 People’s Republic of China; 3 College of Materials Science and Engineering, Chongqing University, Chongqing, 400044 People’s Republic of China; 4 Institute of New Energy Storage Materials and Equipment, Chongqing, 401135 People’s Republic of China; 5 State Key Laboratory of Coal Mine Disaster Dynamics and Control, Chongqing University, Chongqing, 400044 People’s Republic of China

**Keywords:** Electrochemical CO_2_ reduction, Coupled Ag/Sn–SnO_2_ nanosheets, Electronic structure, Porous solid electrolyte, Pure HCOOH solution

## Abstract

Electrocatalytic reduction of CO_2_ converts intermittent renewable electricity into value-added liquid products with an enticing prospect, but its practical application is hampered due to the lack of high-performance electrocatalysts. Herein, we elaborately design and develop strongly coupled nanosheets composed of Ag nanoparticles and Sn–SnO_2_ grains, designated as Ag/Sn–SnO_2_ nanosheets (NSs), which possess optimized electronic structure, high electrical conductivity, and more accessible sites. As a result, such a catalyst exhibits unprecedented catalytic performance toward CO_2_-to-formate conversion with near-unity faradaic efficiency (≥ 90%), ultrahigh partial current density (2,000 mA cm^−2^), and superior long-term stability (200 mA cm^−2^, 200 h), surpassing the reported catalysts of CO_2_ electroreduction to formate. Additionally, in situ attenuated total reflection-infrared spectra combined with theoretical calculations revealed that electron-enriched Sn sites on Ag/Sn–SnO_2_ NSs not only promote the formation of *OCHO and alleviate the energy barriers of *OCHO to *HCOOH, but also impede the desorption of H*. Notably, the Ag/Sn–SnO_2_ NSs as the cathode in a membrane electrode assembly with porous solid electrolyte layer reactor can continuously produce ~ 0.12 M pure HCOOH solution at 100 mA cm^−2^ over 200 h. This work may inspire further development of advanced electrocatalysts and innovative device systems for promoting practical application of producing liquid fuels from CO_2_.

**Supplementary Information:**

The online version contains supplementary material available at 10.1007/s40820-023-01264-6.

## Highlights

- The strongly coupled Ag/Sn–SnO_2_ nanosheets (NSs) were prepared via a versatile electrochemical template strategy, and the generated electron-enriched Sn sites promote the formation of *OCHO and alleviate the energy barriers of *OCHO to *HCOOH.
- Ag/Sn–SnO_2_ NSs afford a superior activity toward CO_2_ electroreduction with current densities up to 2000 mA cm^‒2^ and near-unity selectivity for formate production.
- Ag/Sn–SnO_2_ NSs as the cathode in a membrane electrode assembly with porous solid electrolyte reactor enable the direct production of ~ 0.12 M pure HCOOH solution for 200 h.

**Supplementary Information:**

The online version contains supplementary material available at 10.1007/s40820-023-01264-6.

## Graphical abstract

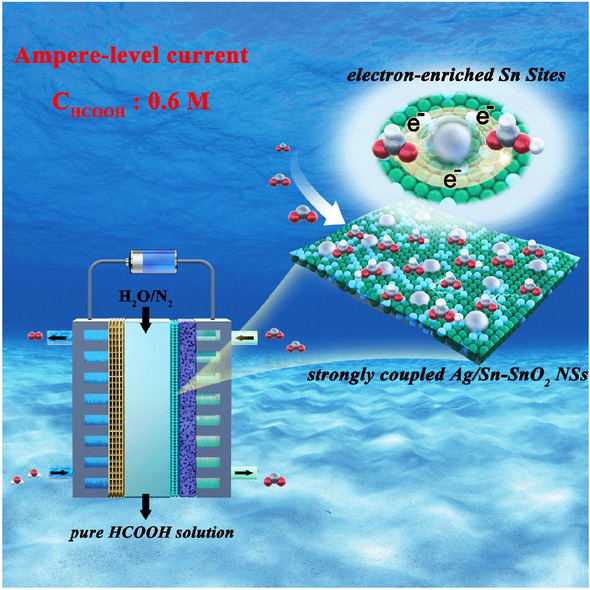

## Introduction

The electrochemical CO_2_ reduction reaction (CO_2_RR) technology provides an elegant route toward a carbon–neutral energy cycle, which addresses the requirements for storage of renewable energy in valuable carbon-based chemicals and fuels [1–3]. Liquid products have a higher volumetric energy density by contrast with gaseous-phase products, enabling them easier to store and distribute [4]. In particular, formate or formic acid (HCOOH) as an important feedstock is widely utilized in pharmaceutical, antiseptics, electrolytic metallurgy, and leather [5]. Unfortunately, to date, the reported electrocatalysts have all failed in commercialization requirements of CO_2_RR with Faradaic efficiency (FE) beyond 90%, partial current density close to 1 A cm^–2^, and long-term operation of at least 100 h [6, 7]. Moreover, the generated liquid product was usually mixed with soluble electrolytes in typical H-type or flow-type reactor [8], requiring extra energy-intensive downstream separation process to purify the liquid fuel solution for practical applications. Thus, it is urgently desirable to explore highly efficient electrocatalysts and develop insoluble electrolyte systems to achieve commercial-grade liquid fuel products.

Over the past decades, Sn-, Bi-, Pb-, and In-based electrocatalysts have been widely exploited for the reduction of CO_2_ to formic acid/formate [9–12]. Among these, Sn-based electrocatalysts are considered as the most promising candidates because of their low toxicity, large abundance, and low cost [13, 14]. However, owing to their medium binding energy to *H derived from HER, CO_2_ conversion over metal Sn is difficult to obtain desirable FE_formate_ [15]. Besides, large overpotential was required to overcome the barrier associated with the initial electron transfer to CO_2_ form the CO_2_^*^ intermediate due to poor electrical conductivity [16, 17]. Recently, it was reported that Sn–SnO_2_ derived from oxidized Sn or reduced SnO_2_ exhibits improved formate selectivity compared to bare Sn, arising from formation of oxygen vacancies or structural defects to enhance the stabilization of CO_2_RR intermediates [18, 19]. Nevertheless, Sn–SnO_2_ catalysts only achieved high FE_formate_ in a narrow potential window and always delivered lower current density.

As known, the product selectivity is mainly related to the electronic configuration of the active sites in electrocatalysts. By reasonably combining foreign metal or non-metal components, it is capable of tailoring interaction between the active sites and intermediates to enhance selectivity [20–23]. Particularly, metal groups as moderator not only optimize the electronic structure of active sites, but also boost significantly its electrical conductivity. As the highest electrical conductivity of all metals, Ag (6.30 × 10^7^ S m^–1^ at 20 ℃) is frequently utilized as a main constituent to increase the electrical conductivity of electrocatalysts [24–28], and its work function of 4.26 eV is also different from both Sn (4.42 eV) and SnO_2_ (5.24 eV) [29–31], which allows the possibility of manipulating the electronic configuration and electrical conductivity of Sn–SnO_2_ spontaneously. Moreover, the increasing number of actives sites, such as the construction of low-dimensional electrodes with more accessibility of catalytic sites, not only enhanced utilization of active centers, but also favored penetration of ions, promoted mass transport of gas, and thus ultimately improved the overall electrocatalytic performance [32, 33]. Consequently, we speculate that assembly of Sn–SnO_2_ units and metal Ag with delicately tuned electronic regulation could generate a significant synergistic effect for an efficient CO_2_RR.

Another prominent challenge that goes beyond the scope of electrocatalysts lies in the liquid products inevitably existing in a mixture with solutes in liquid electrolytes. In a conventional liquid electrolyte, KHCO_3_ or KOH as commonly solutes ensure fast ion conduction between the cathode and anode to obtain low ohmic drop [34]. The budding solid-state batteries adopt ion-conducting solid polymers or ceramics as alternatives of solution electrolyte to assist ions shuttle between anode and cathode [35, 36]. Motivated by this chemical principle, it is urgent to seek innovative electrolytes to replace typical soluble electrolytes, avoid energy-intensive separation, and produce pure fuel solution directly from CO_2_ electrolysers. An integration of rational design in both catalysts and electrolytes, for high activity and pure fuel output, respectively, would help to push the generation of liquid fuels via the CO_2_RR closer to large-scale application.

To support this hypothesis, herein, we designed and synthesized strongly coupled Ag/Sn–SnO_2_ NSs through self-templating transformation and electrochemical reduction strategy. Benefiting from the optimized electronic structure, superior electrical conductivity, and abundant accessible sites, the obtained Ag/Sn–SnO_2_ NSs can accomplish notable performance with an ampere-level current densities (2000 mA cm^–2^) and near-unity selectivity over 90% for CO_2_ electroreduction to formate production, which is much superior to previous reports. Meanwhile, it is also continuous to operate with high FE_formate_ at a current density of 200 mA cm^–2^ for 200 h. The in situ attenuated total reflection-infrared (ATR-IR) spectra and theoretical analysis unraveled that coupling of Ag NPs induces the electronic enrichment of the Sn sites on Sn–SnO_2_ and thereby promotes generation of the crucial *OCHO intermediate and reduces energy barrier of *OCHO to *HCOOH conversion. In addition, to solve the downstream separation cost, porous solid electrolyte (PSE) layer was introduced into a membrane electrode assembly (MEA) reactor, in which the Ag/Sn–SnO_2_ NSs as cathode catalyst can continuously reduce CO_2_ to ~ 0.12 M pure HCOOH solution for 200 h. This work could highlight significant understandings in both the development of advanced catalysts and superior devices for carbon–neutral technologies.

## Experimental Procedures

### Preparation of Electrocatalysts

#### Chemicals and Materials

Tin (II) chloride dihydrate (SnCl_2_·2H_2_O), silver nitrate (AgNO_3_), L-cysteine (C_3_H_7_NO_2_S), 1-methyl-2-pyrrolidinone (NMP), potassium bicarbonate (KHCO_3_), potassium hydroxide (KOH), and ethanol were obtained from Sinopharm Chemical Reagent Co. Ltd. Ag powder, dimethyl sulfoxide-D6 (99.9%, DMSO), deuterium oxide (D_2_O), and formic acid (HCOOH) were purchased from Energy Chemical Co., Ltd. Nafion solution (5 wt%) was obtained from Sigma-Aldrich. All chemical reagents were used directly without further purification. Ultrapure water (> 18.25 M Ω cm) was used for the experiments.

#### Preparation of SnS_2_ NSs

SnS_2_ NSs were synthesized according to the previously reported procedure [37]. In a typical synthesis, 0.228 g of SnCl_2_·2H_2_O and 0.349 g of L-cysteine were dissolved in 60 mL of NMP under magnetic stirring for 3 h. Afterward, the precursor solution was solvothermally reacted at 180 ℃ for 6 h. The product was centrifuged, washed with water and ethanol, and dried at 60 ℃ for 12 h.

#### Preparation of AgSO_4_/SnO_2_ NSs

SnS_2_ NSs as templates were used to fabricate bimetallic oxide nanosheets via cation exchange combined with high-temperature oxidation method. Firstly, 0.1 g of SnS_2_ NSs were dispersed in 80 mL of ethanol with sonication for 30 min. Subsequently, 20 mL of ethanol solution of AgNO_3_ (0.48 mM) was added to the above mixture with magnetic stirring for 12 h at 60 ℃. Finally, the Ag^+^-exchanged SnS_2_ NSs was collected, washed, and dried, and it was further calcined at 500 ℃ for 2 h in O_2_ with a heating rate of 5 ℃ min^–1^.

#### Preparation of Ag/Sn–SnO_2_ NSs

Ag/Sn–SnO_2_ NSs were obtained via an in situ electrochemical self-reconstruction process from AgSO_4_/SnO_2_ NSs in a three-electrode system. Typically, 10.0 mg of AgSO_4_/SnO_2_ NSs was dispersed in the mixture of water, ethanol, and 5 wt.% Nafion solution (8:1:1) with ultrasonic treatment for 30 min and casted onto a 1.0 × 1.0 cm^2^ carbon paper with the mass loading of 2.0 mg cm^–2^. The transformation was carried out in an H-type cell with two compartments separated by a piece of proton exchange membrane (Nafion 117). The working electrode and the reference electrode (saturated Ag/AgCl) were placed in the cathode compartment, and the counter electrode (Pt mesh) was placed in the anodic compartment. Each compartment contained 0.5 M KHCO_3_ electrolyte (15 mL). All potentials were converted to the reversible hydrogen electrode (RHE) reference scale (E_RHE_ = E_Ag/AgCl_ + 0.198 V + 0.0591 V × pH). The cathodic transformation of AgSO_4_/SnO_2_ NSs to Ag/Sn–SnO_2_ NSs was performed via the electrolysis of AgSO_4_/SnO_2_ NSs at ‒1.17 V for 15 min in CO_2_-saturated 0.5 M KHCO_3_ electrolyte.

#### Preparation of Sn–SnO_2_ NSs

Firstly, the obtain SnS_2_ NSs were converted into SnO_2_ NSs at 500 ℃ for 2 h in O_2_ with a heating rate of 5 ℃ min^−1^. Subsequently, the converted SnO_2_ NSs were cathodically reduced to Sn–SnO_2_ NSs, following the similar procedures to Ag/Sn–SnO_2_ NSs.

### Performance Evaluation

#### H-type Cell Measurements

The carbon fiber paper supported Ag/Sn–SnO_2_ (or Sn–SnO_2_) catalyst was directly used as the working electrode in the typical H-type cell with two compartments. The electrolyte was pre-saturated with Ar (pH = 8.4) or CO_2_ (pH = 7.4). A constant CO_2_ flow of 20 sccm was continuously bubbled into the electrolyte to maintain its saturation during CO_2_RR measurements.

#### Flow Cell Measurements

It consisted of a gas diffusion electrode (GDE) loaded with Ag/Sn–SnO_2_ (2.0 mg cm^−2^, 1.0 × 0.5 cm^2^) as the cathode, a piece of bipolar membrane as the separator, and a porous nickel foam as the anode. Ag/AgCl reference electrode was located inside the cathode compartment. During the measurements, CO_2_ gas was directly fed to the cathodic GDE at a rate of 20 sccm. 1.0 M KOH was used as the electrolyte throughout the measurements, which was forced to continuously circulate in the cathode and anode chambers by using peristaltic pumps with 40 mL min^‒1^.

#### Solid Electrolyte Cell Measurements

The two-electrode cells with solid proton conductor were provided by Wuhan Zhisheng New Energy Co., Ltd., and used for pure HCOOH solution production, in which an anion exchange membrane and proton exchange membrane were used for anion and cation exchange, respectively. Around 1 mg cm^−2^ Ag/Sn–SnO_2_ loaded onto the GDL electrode (2.0 cm^2^ electrode area) was used as the cathode, and IrO_2_ was loaded onto a titanium mesh as the anode. The cathode side was supplied with 30 cm^3^ min^–1^ humidified CO_2_ gas, and 0.1 M H_2_SO_4_ aqueous solution was circulated around the anode side with 2 mL min^−1^. Porous styrene–divinylbenzene sulfonated co-polymer was used as the solid ion conductor. Deionized water or humidified N_2_ flow was used to release the HCOOH produced within the solid-state electrolyte layer.

### Computational Methods

All calculations were carried out using Vienna *Ab-initio* Simulation Package (VASP) based on the density functional theory (DFT) [38, 39]. The projector-augmented wave (PAW) with generalized gradient approximation and Perdew–Burke–Ernzerhof functional was used for the electron–ion interactions with a cutoff energy of 500 eV [40, 41]. A vacuum region of 15 Å along the out-plane direction was applied to avoid interactions between the neighboring cells of slab models. Besides, the effect of van der Waals (vdW) interactions was included using the correction scheme of Grimme (D2). According to the experimental characterization results, we constructed model of an Sn–SnO_2_ cluster loaded on Ag (111) to simulate Ag/Sn–SnO_2_. The Bader charge was calculated using the Bader Charge Analysis script by Henkelman and coworkers. The charge transfer from Ag to Sn atom in Sn–SnO_2_ was verified by the Bader charge and differential charge density. The Brillouin zone was sampled on a Monkhorst–Pack k-mesh (1 × 1 × 1 in the structural optimization of Sn–SnO_2_, 3 × 3 × 1 for Ag/Sn–SnO_2_), and the structures were optimized until all the atomic forces have declined below 0.02 eV Å^−1^. The free energy of CO_2_ reduction (Δ*G*) is defined as:

1 $$\Delta G\, = \,\Delta E\, - \,T\Delta S\, + \,\Delta E_{{{\text{ZPE}}}}$$

where *ΔE* denotes calculated total energy change, *T* represents the temperature (298.15 K), *ΔS* is the entropy change, and *ΔE*_ZPE_ is zero-point energy change.

## Results and Discussion

### Synthesis of Strongly Coupled Ag/Sn–SnO_2_ Nanosheets

**Fig. 1:**
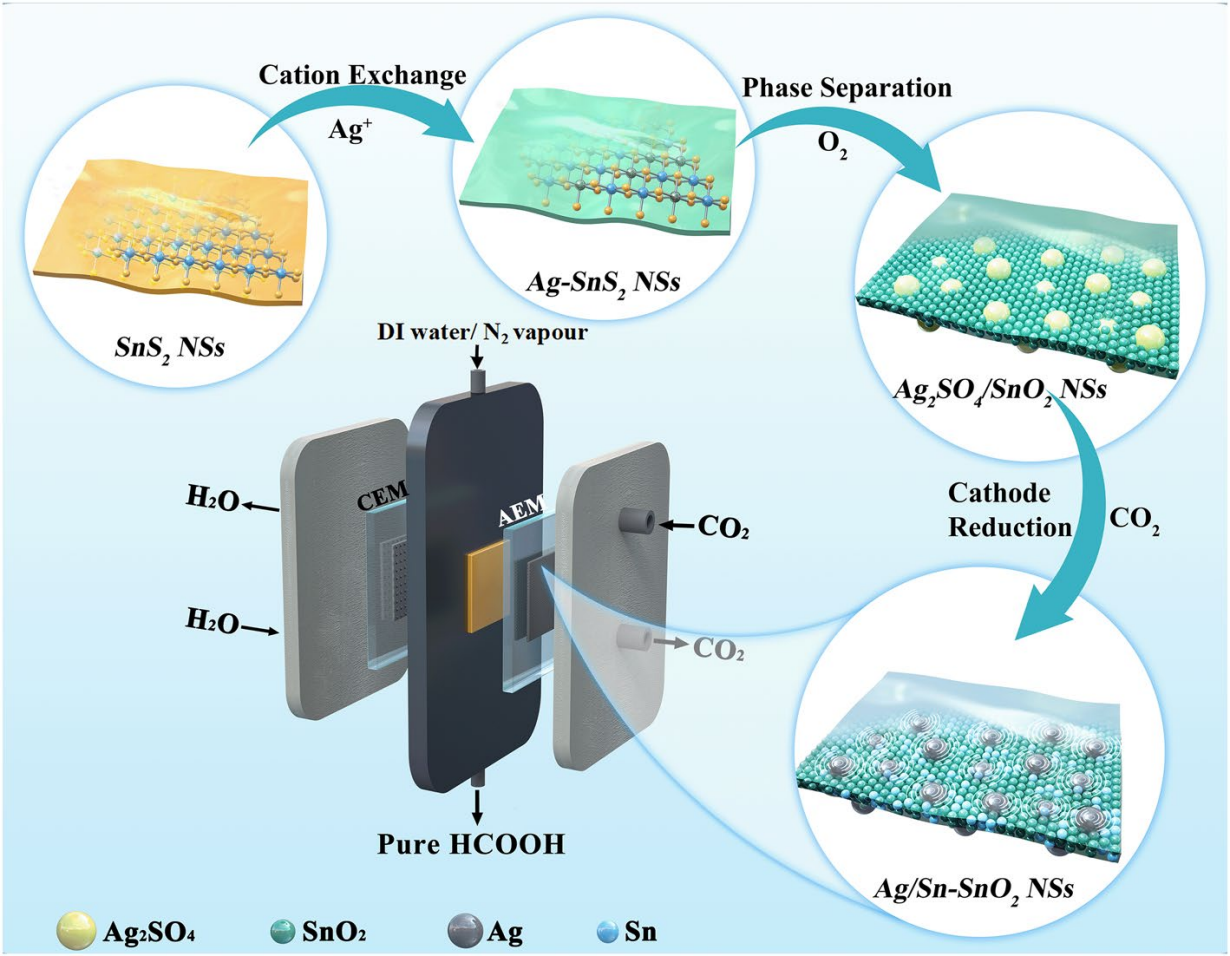
Schematic diagram of the synthesis process of Ag/Sn–SnO_2_ nanosheets for electrocatalytic CO_2_ to pure HCOOH solution

The strongly coupled Ag/Sn–SnO_2_ NSs were engineered via using a versatile electrochemical template strategy (Fig. 1). Here, the SnS_2_ NSs were first prepared according to the solvothermal method (Fig. S1), followed by a facile anion exchange process to obtain silver–tin bimetallic sulfides, designated as Ag–SnS_2_. During this process, the Sn cations from SnS_2_ NSs were partially replaced by Ag^+^ ions due to the smaller solubility product constant of Ag_2_S ($K_{{sp,\;{\text{Ag}}_{2} {\text{S}}}} \le K_{{sp,{\text{ SnS}}_{2} }}$), and their lamellar structure remained almost the same. After calcination treatment in oxygen, the Ag atoms in Ag–SnS_2_ easily escaped from nanosheets, and the coordinated S atoms rapidly formed SO_3_, which aggregated and converted into Ag_2_SO_4_ NPs; meanwhile, Sn atoms kept a lower migration rate inside the nanosheets, and the surrounding S atoms were replaced by O atoms. As a result, the Ag–SnS_2_ NSs evolved into coupled Ag_2_SO_4_/SnO_2_ composites, in which Ag_2_SO_4_ NPs were confined in the nanosheets composed of dense SnO_2_ grains. Finally, the obtained Ag_2_SO_4_/SnO_2_ NSs were converted into Ag/Sn–SnO_2_ NSs under operando CO_2_RR conditions. The coupled Ag/Sn–SnO_2_ NSs were pasted on GDE and fixed in a MEA with PSE layer reactor to achieve a direct and continuous production of pure HCOOH solution.

### Characterizations of Strongly Coupled Ag/Sn–SnO_2_ Nanosheets

**Fig. 2:**
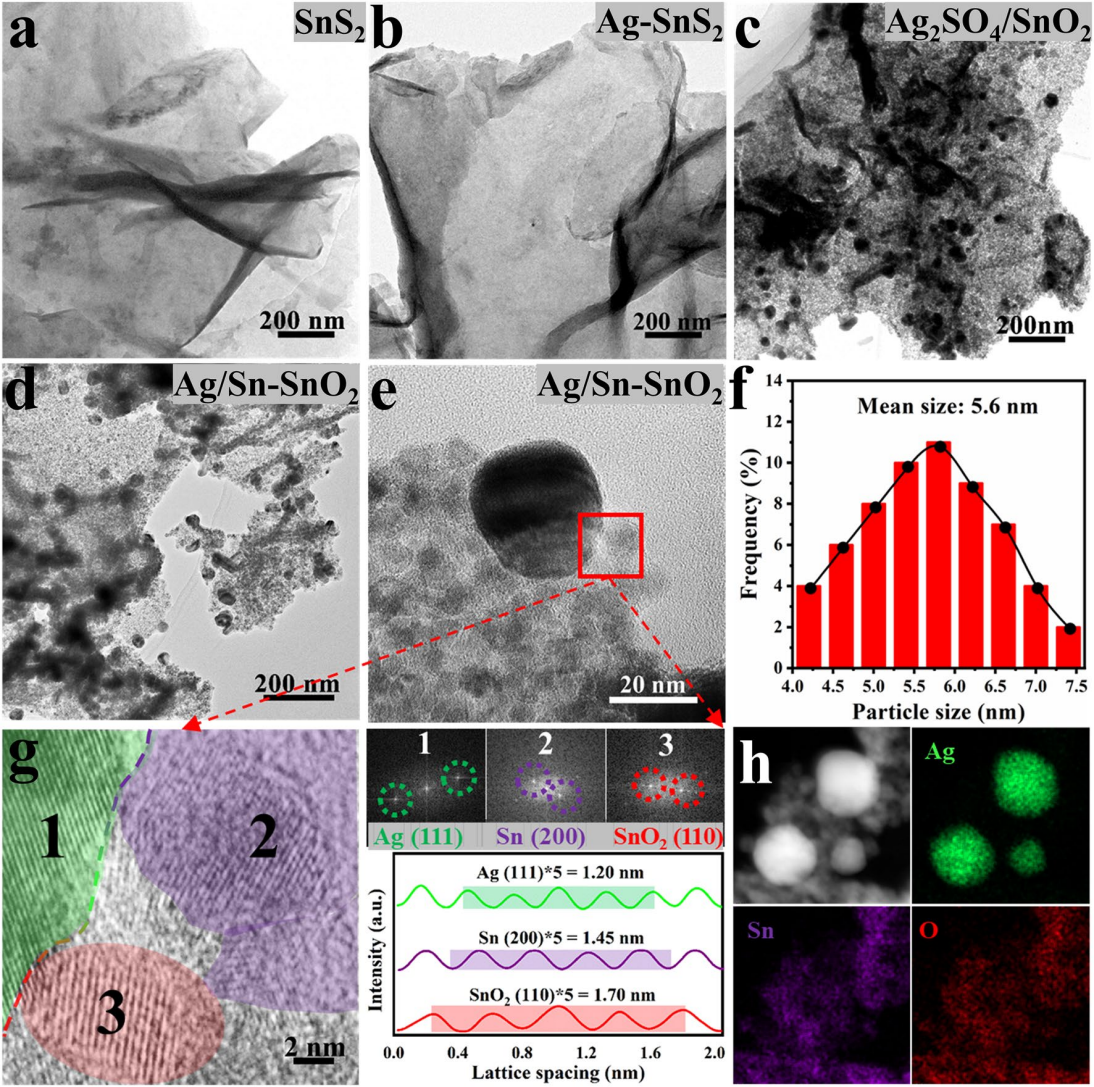
Typical TEM images: **a** SnS_2_ NSs, **b** Ag-SnS_2_ NSs, **c** Ag_2_SO_4_/SnO_2_ NSs, **d** Ag/Sn–SnO_2_ NSs, **e** Ag/Sn–SnO_2_ NSs. **f** Particle size distribution of Sn–SnO_2_ from Ag/Sn–SnO_2_ NSs. **g** HRTEM image and corresponding FFT pattern of Ag/Sn–SnO_2_ NSs, integrated pixel intensities of Ag, Sn, SnO_2_. **h** HAADF-STEM image and EDX mapping images of Ag/Sn–SnO_2_ NSs

The silver–tin bimetallic NSs, synthesized by utilizing SnS_2_ NSs as self-templates, were characterized. Typical transmission electron microscopy (TEM) and high-angle annular dark-field scanning TEM (HAADF-STEM) images (Figs. 2a, b and S2a–d) demonstrate that the morphology of SnS_2_ NSs remains unchanged after Ag^+^ ion-exchange reactions. Energy-dispersive X-ray spectroscopy (EDX) spectrum combined with corresponding mapping images (Fig. S2e, f) confirm that the Ag element was successfully introduced into SnS_2_ NSs with uniform distribution. After the pyrolysis in O_2_ atmosphere, the bimetallic sulfide was transformed into the hybrid Ag_2_SO_4_/SnO_2_. The morphology and structure of the Ag_2_SO_4_/SnO_2_ composite were examined by TEM and high-resolution TEM (HRTEM). As shown in Figs. 2c and S3a, b, some nanoparticles with ~ 10–20 nm in diameter emerge from nanosheets composed of small grains connected each other. Based on the selected area electron diffraction (SAED) pattern (Fig. S3c), two kinds of diffraction rings were found, including the (110), (200), (211), and (301) planes of cubic SnO_2_, and the (200) plane of orthorhombic Ag_2_SO_4_. HRTEM image and corresponding fast Fourier transform pattern reveal that the nanoparticle is Ag_2_SO_4_, which is surrounded by many SnO_2_ grains (Fig. S3d–f). EDX mapping images show these Ag_2_SO_4_ NPs separately embedded into SnO_2_ NSs (Fig. S3g). Besides, their crystal structure and surface statement of Ag_2_SO_4_/SnO_2_ were also further characterized by powder X-ray diffraction (PXRD) and X-ray photoelectron spectroscopy (XPS) technology (Fig. S4).

To obtain Ag/Sn–SnO_2_ NSs, the as-synthesized Ag_2_SO_4_/SnO_2_ precursor was dispersed and pasted on carbon paper and subjected to − 1.8 V versus RHE for 60 min in CO_2_-saturated 0.5 M KHCO_3_ electrolyte. After the electrochemical conversion, the catalyst was collected for further characterization. PXRD pattern unveils that Ag_2_SO_4_ was completely transformed into Ag in the electrocatalysts, while SnO_2_ was partially reduced to Sn metal (Fig. S5). As shown in Fig. 2d, no obvious change in morphology is observed after the electroreduction of Ag_2_SO_4_/SnO_2_ NSs, and the derived Ag NPs are still separately located in the nanosheets that are composed of Sn–SnO_2_ grains with ~ 5.6 nm in size (Fig. 2e, f). HRTEM image (Fig. 2g) distinctly shows a region of interfacial contact among three different crystal planes with lattice spacings of about 0.24, 0.29 and 0.34 nm, belonging to the (111) plane of Ag, the (200) plane of Sn, and the (110) plane of SnO_2_, respectively. The derived Ag/Sn–SnO_2_ NSs possess intimate interfaces of Ag and Sn–SnO_2_, which was also confirmed by corresponding EDX mapping images and EDX line scans (Figs. 2h and S6). The Sn–SnO_2_ NSs were also prepared for comparison through the similar procedure (Figs. S7 and S8). Further insight into the chemical states of Ag/Sn–SnO_2_ NSs was obtained by XPS. As shown in Fig. S9a, Ag 3*d* spectrum for Ag/Sn–SnO_2_ NSs fits well with two pairs of characteristic peaks at 368.1 and 373.9 eV, and 367.6 and 373.6 eV, which can be assigned to metal Ag and Ag^+^, respectively [42]. As for Sn 3*d* spectrum in Fig. S9b, the two sets of doublet peaks for Ag/Sn–SnO_2_ at the binding energy of 484.9 and 494.2 eV together with 486.1 and 494.8 eV ascribe to metal Sn and Sn^4+^, respectively [43]. Obviously, the binding energy of Sn 3d in Ag/Sn–SnO_2_ NSs negatively shifted 0.15 eV compared to that of Sn–SnO_2_ NSs, indicating the electronic interaction between the Ag NPs and the Sn–SnO_2_ NSs.

**Fig. 3:**
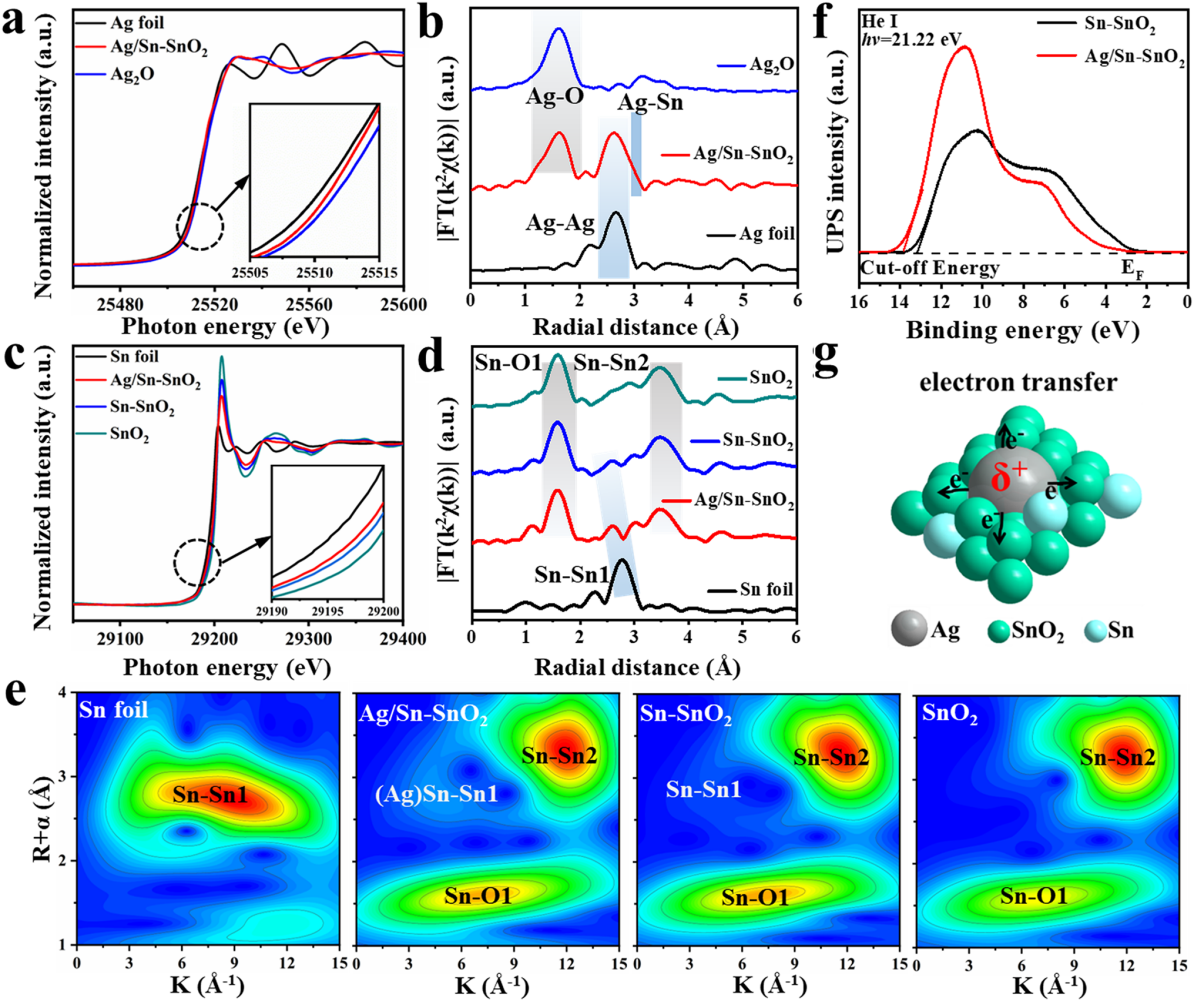
Fine-structure characterization: **a** Ag K-edge XANES spectra, **b** Ag K-edge extended XANES oscillation functions K^3^χ(k), **c** Sn K-edge XANES spectra, **d** Sn K-edge extended XANES oscillation functions K^3^χ(k), **e** Wavelet transform EXAFS of Sn of Sn foil, Sn–SnO_2_, Ag/Sn–SnO_2_, and SnO_2_. **f** UPS spectra of Ag/Sn–SnO_2_ and Sn–SnO_2_. **g** Schematic illustration of electron transfer at Ag/Sn–SnO_2_ NSs

In order to deeply investigate the effect of Ag NPs on the electronic and coordination structure of Sn–SnO_2_, X-ray absorption near-edge structure (XANES) was carried out. The XANES spectra of the Ag K-edge in different samples (Fig. 3a) show that the adsorption edges of Ag/Sn–SnO_2_, Ag_2_O, and Ag foil are very close to each other. The magnified image (inset in Fig. 3a) shows that Ag/Sn–SnO_2_ exhibits a tendency for the pre-edge feature to shift to Ag_2_O, compared with the Ag foil. The K^3^-weighted EXAFS spectra of Ag/Sn–SnO_2_ in Fig. 3b show that the two main peaks correspond to Ag–O and Ag–Ag (Ag-Sn) bonds compared to Ag_2_O and Ag foil. It is worth noting that the interatomic distance of the Ag–Ag bond (2.92 Å) in Ag/Sn–SnO_2_ is slightly longer than that of Ag foil (2.86 Å) due to the formation of Ag-Sn bonding, which is further verified by the Ag K-edge EXAFS fitting results displayed in Figs. S10 and S11 and Table S1. In the XANES spectra of the Sn K-edge (Fig. 3c), the edge position of Sn–SnO_2_ and Ag/Sn–SnO_2_ located between those of Sn foil and SnO_2_, and Ag/Sn–SnO_2_ shifted to lower energy compared with Sn–SnO_2_, suggesting their decreased congruity in the coordination environment. To further illustrate the fine structure of Ag/Sn–SnO_2_, their XANES spectra are compared and shown in Fig. 3d, in which the peaks at 1.34 and 2.42 Å are attributed to the Sn–O bond and Sn–Sn (Sn–Ag) interaction, respectively. A slight compression of the Sn–Sn bond derives from the difference between the atomic radii of Sn and Ag, further confirming their successful alloying. The wavelet transform analysis also confirmed the different coordination of Sn atom between the Sn–SnO_2_ and Ag/Sn–SnO_2_ samples (Fig. 3e). According to the Sn K-edge EXAFS fitting curves (Table S2), it is noticed that the average coordination numbers of Sn–O (3.7), Sn–Ag (2.0), and Sn–Sn (2.1) in Ag/Sn–SnO_2_ are obviously less than those of Sn–O (4.7) and Sn–Sn (3.1) in Sn–SnO_2_, implying the emergence of coordinatively unsaturated Sn sites after the coupling of Ag. Usually, these unsaturated metal atoms would cause structural distortions or defects, leading to greatly modified electronic properties. The work functions of Ag/Sn–SnO_2_ and Sn–SnO_2_ were further investigated to clarify the difference in their electronic properties by ultraviolet photoelectron spectroscopy (UPS). Sn in Sn–SnO_2_ is easily oxidized back to SnO_2_ during the UPS preparation, so that the work function of Sn–SnO_2_ (5.17 eV) does not obviously decrease compared to that of SnO_2_. In contrast to transition metal, noble metal Ag is difficult to be oxidized, and the coupled Ag/Sn–SnO_2_ displays a markedly lower work function (4.73 eV). Thus, Ag NPs would transfer electrons to Sn–SnO_2_, which is further confirmed by the cyclic voltammetry (CV) test (Fig. S13), inducing an upshift of the Fermi level of Ag/Sn–SnO_2_ (Fig. 3f). Briefly, it is concluded that the introduction of Ag into Sn–SnO_2_ facilitates the electron transfer from Ag atoms to Sn atoms in Sn–SnO_2_, leading to electron enrichment in Sn–SnO_2_ surfaces (Fig. 3g).

### Electrocatalytic CO_2_RR Performances in Typical Reactor

**Fig. 4:**
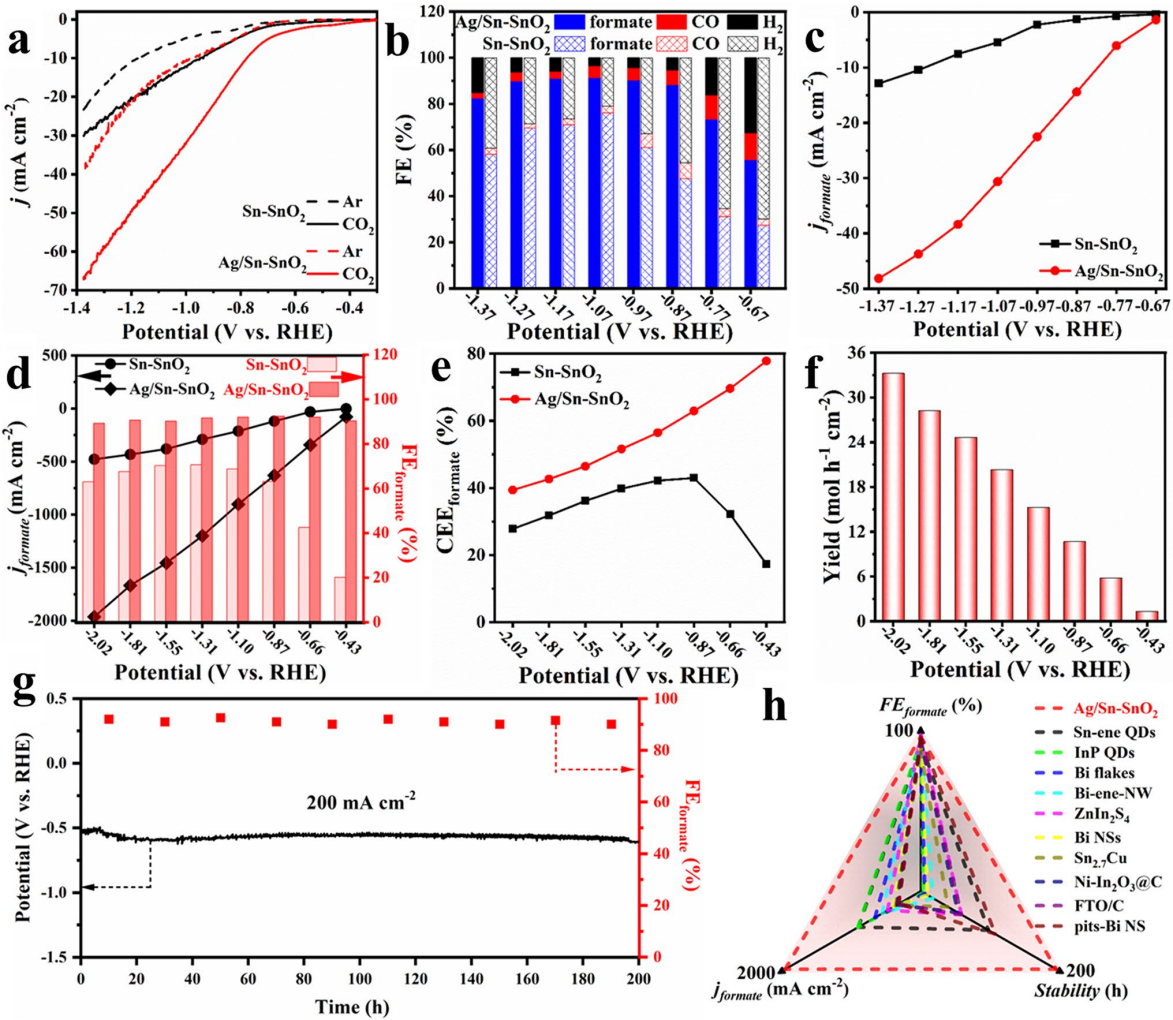
Electrocatalytic CO_2_RR performance in typical reactors:** a** LSV curves, **b** FE of formate CO and H_2_, **c** partial current densities of formate of Sn–SnO_2_ NSs and Ag/Sn–SnO_2_ NSs of H-type reactor in 0.5 M KHCO_3_; **d** FE of formate and partial current densities of formate of Sn–SnO_2_ NSs and Ag/Sn–SnO_2_ NSs, **e** cathodic energy efficiency of formate of Sn–SnO_2_ NSs and Ag/Sn–SnO_2_ NSs, **f** yield of formate over Ag/Sn–SnO_2_ NSs, **g** long-term amperometric stability of Ag/Sn–SnO_2_ NSs, **h** comparison of stability, FE of formate, and current density with those of reported Sn-based catalysts of Flow-type reactor

The catalytic performance of Ag/Sn–SnO_2_ NSs for CO_2_ electroreduction was evaluated in an H-type reactor using 0.5 M KHCO_3_ as electrolyte. As shown in Fig. 4a, the current densities of Sn–SnO_2_ NSs are enhanced with the combination of Ag NPs, and it exhibits considerable current densities of 69 mA cm^−2^ compared to Sn–SnO_2_ NSs with 29.5 mA cm^−2^ at –1.37 V. The promotion of current density by Ag coupling was further confirmed by the linear scanning voltammetry (LSV) curves in Ar-saturated KHCO_3_ electrolyte. The products of CO_2_RR were quantitatively analyzed at different potentials between − 0.67 and − 1.37 V over Sn–SnO_2_, and Ag/Sn–SnO_2_ NSs (Figs. S14–S16). Figure 4b shows that the FE_formate_ of Ag/Sn–SnO_2_ NSs can remain above 90% in a wide range of potentials from –0.87 to –1.37 V with the maximum value of ~ 93% at − 1.17 V, which is 1.2 times larger than that of Sn–SnO_2_ NSs (78%). When bare Ag NPs were utilized as electrocatalysts, only conversion of CO_2_ toward CO was achieved (Fig. S16). We further calculated the partial current density of formate (*j*_formate_) for Ag/Sn–SnO_2_ and Sn–SnO_2_ NSs at all of applied potentials. As shown in Fig. 4c, Ag/Sn–SnO_2_ is capable of delivering a *j*_formate_ of about 48.5 mA cm^−2^ at − 1.37 V, which is about 3 times higher than that of Sn–SnO_2_ (16 mA cm^−2^). Besides, the effect of Ag content in Ag/Sn–SnO_2_ NSs on the performance of CO_2_ reduction was also investigated (Fig. S17). With the increase of Ag content, the current density increases from 30 to 67 mA cm^−2^ and then decrease to 58 mA cm^−2^. The corresponding FE_formate_ also follows volcanic-like trend at the applied potential of − 1.27 V, which may be due to that part of the Ag, which is not in close contact with Sn–SnO_2_, tends to reduce CO_2_ to CO.

To decipher the origin of high activities of Ag/Sn–SnO_2_ NSs, their double layer capacitances (C_dI_) were estimated according to corresponding electrochemically active surface areas (Fig. S18a). The C_dI_ of Ag/Sn–SnO_2_ and Sn–SnO_2_ NSs were calculated to be 1.62 and 0.66 mF cm^−2^, respectively, illustrating that the Ag/Sn–SnO_2_ NSs can provide more active sites. The C_dI_-normalized *j*_formate_ value of Ag/Sn–SnO_2_ NSs was larger than that of Sn–SnO_2_ NSs (Fig. S18b), elucidating Sn–SnO_2_ NSs with improved intrinsic activity by incorporation of Ag NPs. To gain insight into the reaction kinetics, the Tafel slopes of the catalysts were plotted in Fig. S18c. The Tafel slope of Ag/Sn–SnO_2_ NSs (107.2 mV dec^–1^) is lower than that of Sn–SnO_2_ NSs (172.8 mV dec^–1^), indicating the favorable kinetic activity for the electrocatalytic reduction of CO_2_ to formate over Ag/Sn–SnO_2_ NSs. Moreover, multi-step proton–electron coupling is involved in the reduction of CO_2_, and the charge transfer process from catalysts to intermediates was also investigated by the electrochemical impedance spectroscopy (EIS). By contrast, Ag/Sn–SnO_2_ NSs have a smaller impedance value of ~ 10 Ω compared to Sn–SnO_2_ NSs (~ 60 Ω) (Fig. S18d), implying good electrical conductivity, which confirms that after combination with Ag, the charge transfer resistance of Sn–SnO_2_ is significantly decreased and thus promotes the reaction kinetics. Furthermore, the oxidative LSV scans under Ar-bubbled KOH electrolyte further displayed that the potential for OH^–^ adsorption on the Ag/Sn–SnO_2_ NSs surface was more negative than that of Sn–SnO_2_ NSs, suggesting its most powerful ability to stabilize the CO_2_*^−^ intermediate (Fig. S19). To the best of our knowledge, Ag/Sn–SnO_2_ NSs exceed previously reported Sn-based electrocatalysts for formate production by CO_2_RR in H-type reactor (Fig. S20 and Table S3). In addition, Ag/Sn–SnO_2_ NSs also exhibits good stability with high FE_formate_ during a 25-h potentiostatic test (Fig. S21). After the long-term test, no obvious changes in crystallinity, surface statement, and morphology for the Ag/Sn–SnO_2_ NSs are observed (Fig. S22), elucidating their good activity and stability.

Gas diffusion electrode (GDE) can overcome mass-transfer limitation of CO_2_ due to its low solubility in aqueous solution and promotes the realization of the industrial-level current density, and the CO_2_RR performance of Ag/Sn–SnO_2_ NSs was further measured in a flow-type reactor (Fig. S23). Figures 4d and S24 show that Ag/Sn–SnO_2_ NSs gave above 90% FE_formate_ over a wide potential window from –0.43 to –2.02 V, while Sn–SnO_2_ delivered lower formate selectivity as it failed to suppress competitive HER. By contrast, Ag/Sn–SnO_2_ NSs achieved an ultrahigh formate partial current density of 2000 mA cm^–2^ at –2.01 V, outperforming most of the advanced electrocatalysts for formate generation by CO_2_RR (Figs. 4d and S24f). In the meantime, Ag/Sn–SnO_2_ NSs manifested a higher cathodic energy efficiency (CEE) than that of Sn–SnO_2_ for reduction of CO_2_ to formate (Fig. 4e). To further intuitively evaluate the efficiency of Ag/Sn–SnO_2_ NSs, the corresponding formate yield at the selected potentials per unit catalyst load area and time was also calculated. As shown in Fig. 4f, the yield of formate for Ag/Sn–SnO_2_ NSs increased with the increase of applied potential, and the value reached 33.5 mol h^–1^ cm^–1^ at 2.02 V. Moreover, the long-term stability a CO_2_RR electrocatalyst is another prerequisite for its practical implementation. Correspondingly, the constant-current electrolysis of Ag/Sn–SnO_2_ NSs at the current density of 200 mA cm^–2^ in the flow-type reactor was operated and is displayed in Fig. 4g. Clearly, no obvious increase in potential was noticed after 200 h continuous electrolysis. Meanwhile, an excellent formate selectivity was well maintained, illustrating the industrial superiority and feasibility of the Ag/Sn–SnO_2_ NSs at such a large current density. Such remarkable catalytic performance of Ag/Sn–SnO_2_ NSs is the best one among those reported catalysts of CO_2_ electroreduction to formate (Fig. 4h and Table S4).

### In Situ Characterizations and Theoretical Calculations

**Fig. 5:**
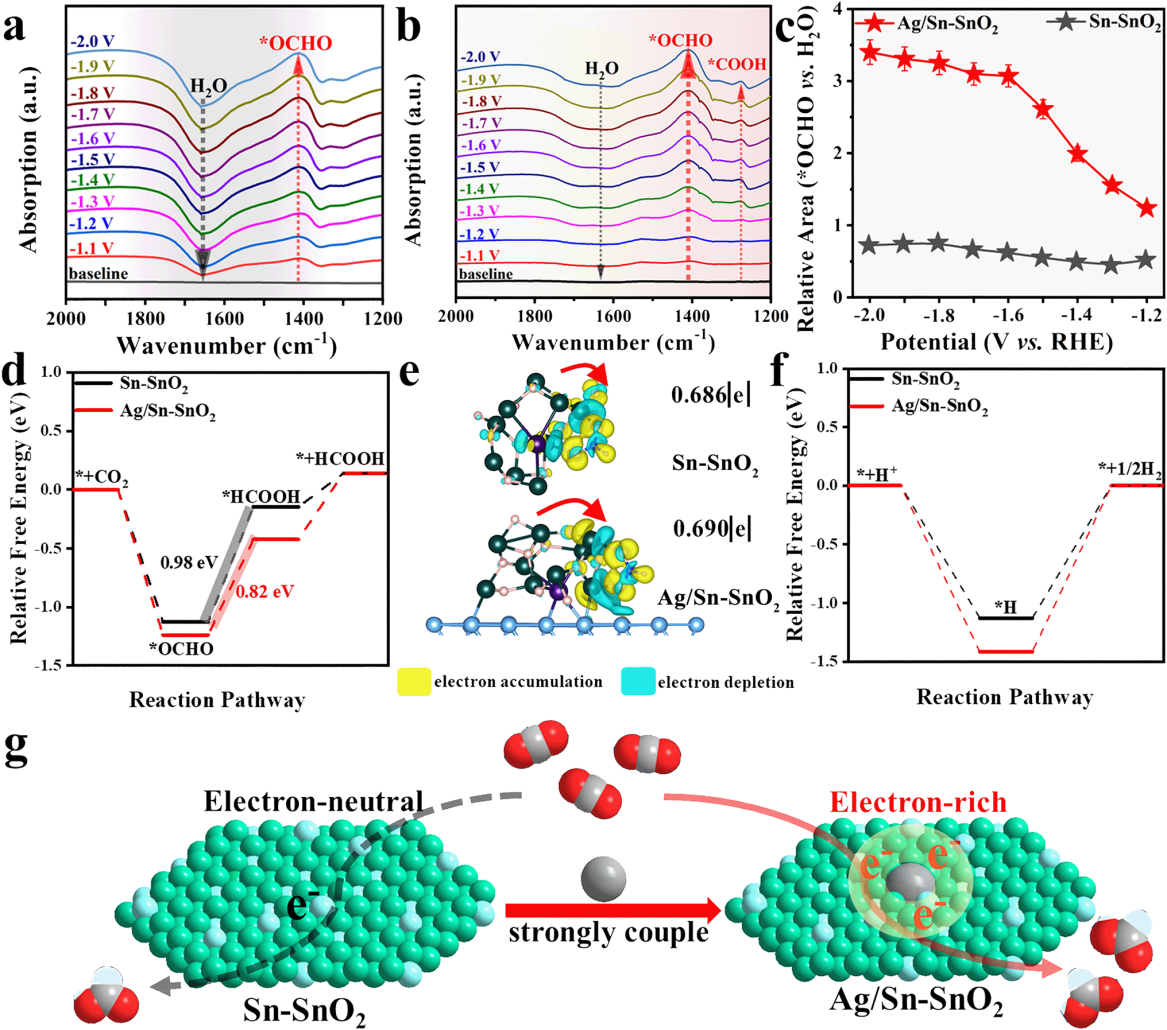
Operando ATR-IR spectra collected at different applied potentials in CO_2_-saturated 0.5 M KHCO_3_:** a** Sn–SnO_2_, **b** Ag/Sn–SnO_2_, **c** comparison of the relative peak area ratio of *OCHO/H_2_O. Theoretical calculations for Ag/Sn–SnO_2_ and Sn–SnO_2_: **d** free energy profiles of CO_2_ reduction to HCOOH,** e** differential charge density plot of the *OCHO intermediate adsorption structure, **f** free energy profiles of H_2_ evolution. **g** Schematic diagram of CO_2_RR over electron-rich Sn sites and electron-neutral Sn sites

To further understand the electroreduction of CO_2_ to formate process, operando ATR-IR spectra were performed to monitor the intermediates. As shown in Fig. 5a, an upward peak at 1401 cm^–1^ is assigned to the *OCHO group [9, 10], and another downward peak at 1630 cm^−1^ is ascribed to interfacial H_2_O [44, 45], implying that the *OCHO intermediate was generated while the absorbed H_2_O molecules were participated for Sn–SnO_2_. Remarkably, the consumption of interfacial H_2_O is equivalent to the formation of *OCHO, implying that the reduction of CO_2_ is accompanied by significant H_2_ evolution. By integrated Ag NPs in Sn–SnO_2_ NSs, the consumption of absorbed H_2_O characteristic band is obviously suppressed (Fig. 5b), and corresponding relative peak area ratio of *OCHO/H_2_O for Ag/Sn–SnO_2_ is bigger than Sn–SnO_2_ (Fig. 5c), which clarifies that Ag/Sn–SnO_2_ can effectively weaken the formation of H_2_ and promote the reduction of CO_2_. A weak peak belonging to *COOH group was observed at 1268 cm^−1^ [46], because the uncovered Ag in Ag/Sn–SnO_2_ NSs prefer to bond the formation of CO intermediate (Fig. 5b). Based on the analysis of electrochemical in situ FTIR results, it is likely that CO_2_ reduction follows the reaction pathway listed as follows:

2 $${\text{CO}}_{2} + ^{*} \to {\text{CO}}_{2} ^{*}$$

3 $${\text{CO}}_{{2}}^{*} + {\text{ e}}^{-} + {\text{H}}^{ + } \to ^{*}{\text{OCHO}} + {\text{CO}}_{{3}}^{{{2}{-}}}$$

4 $$\phantom{i}^{*}{\text{OCHO}} + {\text{H}}^{ + } + {\text{e}}^{ - } \to ^{*}{\text{HCOOH}}$$

5 $$\phantom{i}^{*}{\text{HCOO}} \to {\text{HCOO}}^{-} + ^{*}$$

First-principles calculations were carried out to gain insights into high performance of CO_2_ electroreduction over Ag/Sn–SnO_2_ NSs. The Ag/Sn–SnO_2_ slab was modeled by optimizing the Sn–SnO_2_ grains on the surface of Ag NPs (Fig. S25), and corresponding Gibbs free energy for each reaction step of CO_2_RR process was calculated (Fig. S26). As shown in Fig. 5d, the formation of intermediate HCOO* is an exothermic process, and Ag/Sn–SnO_2_ occupies obviously lower key *OCHO intermediate formation energy compared to Sn–SnO_2_. The transformation of *OCHO to *HCOOH is the most endothermic on both systems and uphill energy on Ag/Sn–SnO_2_ is smaller than that on Sn–SnO_2_ (0.82 vs. to 0.98 eV), indicating that HCOOH formation on Ag/Sn–SnO_2_ is more favorable. Subsequently, the desorption of *HCOOH only needs to overcome the smaller energetic barriers for both, illustrating that the second proton-coupled electron step dominates the CO_2_ reduction process. Charge density difference plots of OCHO adsorption on Ag/Sn–SnO_2_ and Sn–SnO_2_ are presented in Fig. 5e. Notably, charge redistribution over Ag/Sn–SnO_2_ is the more significant, rationalizing the stronger adsorption. Moreover, Bader charge analysis reveals that the *OCHO species on Ag/Sn–SnO_2_ obtains a charge of 0.690 |e|, which is greater than that (0.686 |e|) on Sn–SnO_2_, indicating that the electrons of Sn atoms in Ag/Sn–SnO_2_ are easily transferred to *OCHO. In addition, as main side reaction, HER was also studied (Fig. S27), and the free energy profiles are displayed in Fig. 5f. The uphill free energy of the desorption of *H to H_2_ on Ag/Sn–SnO_2_ surface is much higher than that on Sn–SnO_2_ (Fig. 5f), suggesting the superior inhibition of H_2_ production. Overall, both experimental and theoretical results elucidate that the coupled Ag/Sn–SnO_2_ with electron-rich Sn sites not only facilitates the successive hydrogenation of *CO_2_ to *OCHO to *HCOOH, but also further hinders H_2_ formation, thus resulting in enhancement selectivity of formate in a wide range of potential (Fig. 5g).

**Fig. 6:**
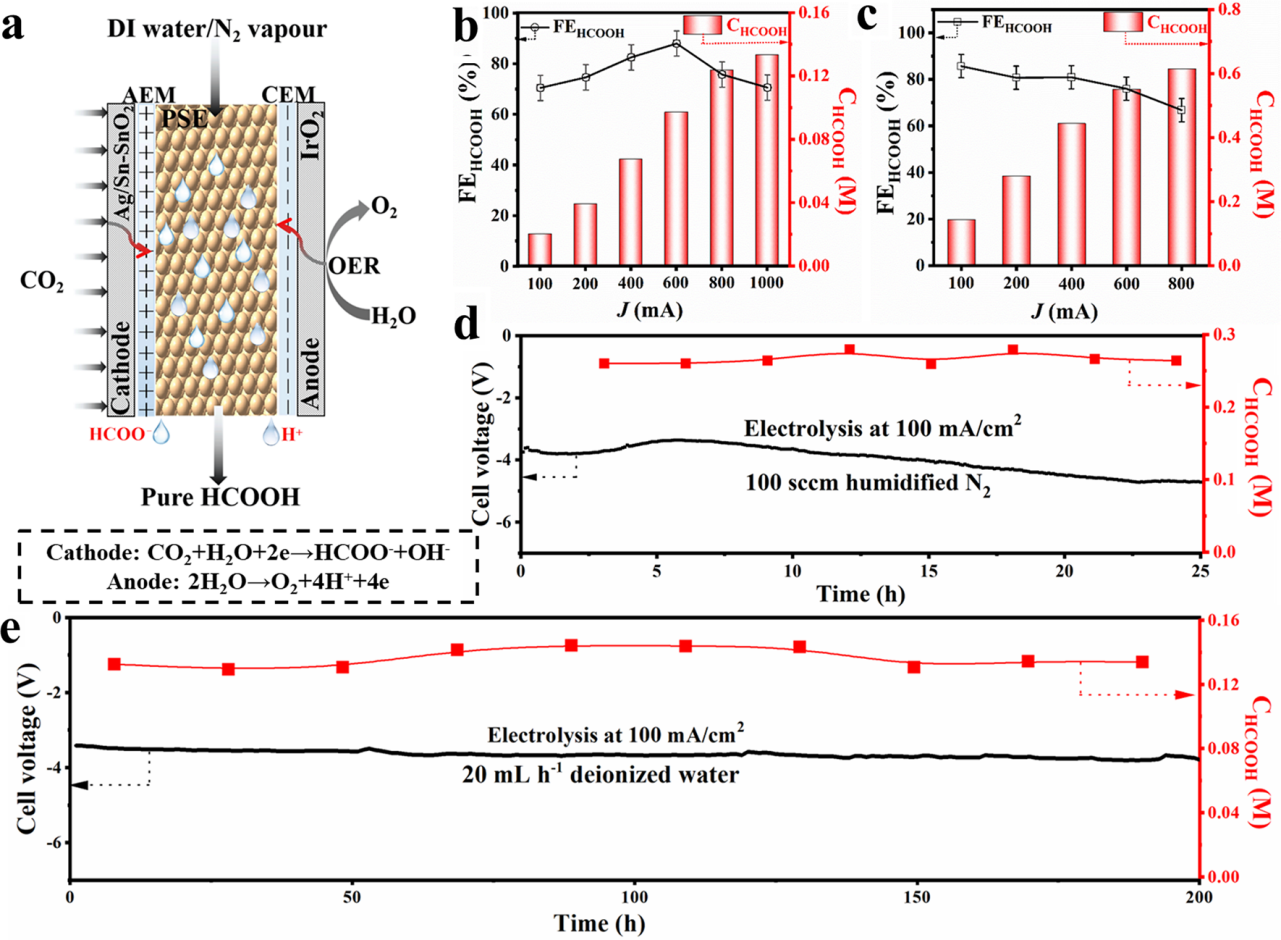
Electrocatalytic CO_2_RR performance of Ag/Sn–SnO_2_ in a 2 cm^2^ MEA with PSE layer reactor:** a** schematic illustration of CO_2_ reduction to pure HCOOH with OER reaction in the PSE-cell (The pure HCOOH product can be formed via the ionic recombination of crossed HCOO^−^ and H^+^ ions in the PSE layer, and diffuse away through DI water or N_2_ vapor flow). Concentration of HCOOH under different cell currents, along with the corresponding FE_HCOOH_, **b** DI water as diffuse carrier with flow rate of 60 mL h^−1^, **c** humidified N_2_ as diffuse carrier with flow rate of 100 sccm. Long-term electrolysis under a current density of 100 mA cm^−2^, **d** humidified N_2_ as diffuse carrier with flow rate of 100 sccm, **e** DI water as diffuse carrier with flow rate of 20 mL h^−1^

Up to now, although formate can be exclusively generated via a CO_2_RR approach with high-performance electrocatalysts, the as-obtained products were in low concentrations actually and mixed with solute salts, which requires extra separation and concentration treatments to obtain pure liquid fuel for subsequent applications. To promote large-scale application of producing liquid fuels from CO_2_RR pathway, porous solid electrolytes (PSE) is employed to replace water-soluble electrolytes and achieve direct synthesis of pure liquid acid solution. PSE layer can efficiently deliver ions between the cathode and anode, but does not introduce additional solutes to mix with produced HCOOH. As shown in Figs. 6a and S28, the cathode and anode of PSE layer are Ag/Sn–SnO_2_-coated GDE and Ti mesh-supported IrO_2_, which sandwiched cation exchange membranes (CEMs) and anion exchange membranes (AEMs), respectively. The cathode side was uninterruptedly supplied with humidified CO_2_ flow for CO_2_RR, and generated HCOO^−^ ions on Ag/Sn–SnO_2_ were driven to pass through the AEM and arrive the middle PSE layer by the electrical field. Meanwhile, the anode side was circulated with 0.1 M H_2_SO_4_ solution for water oxidation, and H^+^ generated on IrO_2_ moved through the CEM into the middle PSE layer. In the middle chamber, a PSE layer facilitated ion transportation with minimized ohmic losses, and the HCOOH molecules formed via the ionic recombination were then efficiently brought out through this porous layer via the flow of deionized (DI) water or N_2_ vapor.

The LSV curve of CO_2_RR in a 2 cm^2^ Ag/Sn–SnO_2_//PSE//IrO_2_ cell plotted in Fig. S29a shows that the overall current density gradually increases as the cell voltage ramps up. The selectivity of HCOOH in the PSE-cell follows volcanic-like trend and max FE_HCOOH_ reaches up to 83% (Figs. 6b and S29b), but their value is lower compared to results of typical reactors, which rooted in the different pH (acidic pH in MEA with PSE and neutral/alkaline pH in H-type/flow-type reactor, respectively) and the liquid product distribution. With the increase of current density, corresponding concentration of HCOOH solution varies from 15 to 130 mM at this deionized (DI) water flow rate (Fig. 6b), and maximum partial current density is 350 mA cm^−2^ (Fig. S29c). To further improve concentration of the generated HCOOH, humidified N_2_ was employed to replace the DI water to carry HCOOH molecules in PSE layer. As shown in Fig. S29d, the overall current density also reaches 1000 mA with a fixed N_2_ flow rate of 100 sccm. It is observed in Figs. 6c and S29e that FE_HCOOH_ also follows a trend of rising up first and then falling down, but the concentration of HCOOH is significantly improved under the same current, and corresponding current density can reach 270 mA cm^−2^ (Fig. S29f).

In view of the advantage of producing high-concentration HCOOH solution with humidified N_2_ flow, a long-term electrolysis experiment was also implemented to realize the continuous production of pure HCOOH solution. However, it was noticed that the cell voltage was apparently increased to maintain the current of 200 mA as the electrolysis time was prolonged (Fig. 6d), which is mainly attributed to the gradual degradation of membranes and solid electrolytes without enough wetting conditions [47]. Because it is difficult to prevent the degradation of AEM on the current technical level, DI water as a substitute was utilized to explore the durability of Ag/Sn–SnO_2_ for long-term operation at the same current for reduction of CO_2_ to pure HCOOH with a lower flow rate of 20 mL h^−1^. Strikingly, a stable production of ~ 0.1 M pure HCOOH was achieved at a large current density of 100 mA cm^−2^ during 200 h continuous electrolysis periods (Fig. 6e). Such unparalleled activity, yield, and excellent long-term stability for reduction of CO_2_ to pure HCOOH surpass previous reports (Table S5). Consequently, the exceptional results of Ag/Sn–SnO_2_ present promising prospect of commercial production of pure HCOOH from CO_2_.

## Conclusions

In conclusion, we have developed strongly coupled Ag/Sn–SnO_2_ NSs by combined self-templating transformation and electrochemical reduction strategy. On account of its regulated electron configuration and superior electrical conductivity, the Ag/Sn–SnO_2_ NSs with ample accessible sites as electrocatalysts are capable of exhibiting a combination of high activity and stability in the CO_2_-to-formate reduction exceeding 200 h of continuous operation, outperforming most of reported Sn-based electrocatalysts. Moreover, operando ATR-IR spectroscopy and theoretical calculations reveal that the incorporation of Ag NPs into Sn–SnO_2_ NSs can promote the formation of the *OCHO, alleviate the energy barriers of *OCHO to *HCOOH, and suppress the competitive H_2_ production. Additionally, Ag/Sn–SnO_2_ NSs as the cathode in a MEA with PSE reactor enable the direct production of ~ 0.12 M pure HCOOH solution for 200 h. The momentous achievements in this work would be indicative to integrating the electrode materials and electrolytic systems for accelerating the practical application of carbon–neutral technologies.

## Supplementary Information

Below is the link to the electronic supplementary material.

Supplementary file1 (PDF 3577 KB)

## References

[1] J. Ma, X. Xiong, D. Wu, Y. Wang, C. Ban et al., Band position-independent piezo-electrocatalysis for ultrahigh CO_2_ conversion. Adv. Mater. **35**, 2300027 (2023). 10.1002/adma.202300027 10.1002/adma.202300027 36876444

[2] B. Yang, K. Liu, H. Li, C. Lui, J. Fu et al., Accelerating CO_2_ electroreduction to multicarbon products via synergistic electric–thermal field on copper nanoneedles. J. Am. Chem. Soc. **144**, 3039–3049 (2022). 10.1021/jacs.1c11253 35112839 10.1021/jacs.1c11253

[3] Q. Wang, M. Dai, H. Li, Y. Lu, T. Chan et al., Asymmetric coordination induces electron localization at ca sites for robust CO_2_ electroreduction to CO. Adv. Mater. **35**, 2300695 (2022). 10.1002/adma.202300695 10.1002/adma.202300695 36929182

[4] H. Shin, K.U. Hansen, F. Jiao, Techno-economic assessment of low-temperature carbon dioxide electrolysis. Nat. Sustain. **4**, 911–919 (2021). 10.1038/s41893-021-00739-x

[5] J. Fan, X. Zhao, X. Mao, J. Xu, N. Han et al., Large-area vertically aligned bismuthene nanosheet arrays from galvanic replacement reaction for efficient electrochemical CO_2_ conversion. Adv. Mater. **33**, e2100910 (2021). 10.1002/adma.202100910 34302394 10.1002/adma.202100910

[6] M. Zhong, K. Tran, Y. Min, C. Wang, Z. Wang et al., Accelerated discovery of CO_2_ electrocatalysts using active machine learning. Nature **581**, 178–183 (2020). 10.1038/s41586-020-2242-8 32405017 10.1038/s41586-020-2242-8

[7] F.P. García de Arquer, C.T. Dinh, A. Ozden, J. Wicks, C. McCallum et al., CO_2_ electrolysis to multicarbon products at activities greater than 1 A cm^−2^. Science **367**, 661–666 (2020). 10.1126/science.aay4217 32029623 10.1126/science.aay4217 PMC13531662

[8] C. Xia, P. Zhu, Q. Jiang, Y. Pan, W. Liang et al., Continuous production of pure liquid fuel solutions via electrocatalytic CO_2_ reduction using solid-electrolyte devices. Nat. Energy **4**, 776–785 (2019). 10.1038/s41560-019-0451-x

[9] M. Zhang, S. Zhou, W. Wei, D.-D. Ma, S.-G. Han et al., Few-atom-layer metallene quantum dots toward CO_2_ electroreduction at ampere-level current density and Zn-CO_2_ battery. Chem Catal. **2**, 3528–3545 (2022). 10.1016/j.checat.2022.10.001

[10] M. Zhang, W. Wei, S. Zhou, D.-D. Ma, A. Cao et al., Engineering a conductive network of atomically thin bismuthene with rich defects enables CO_2_ reduction to formate with industry-compatible current densities and stability. Energy Environ. Sci. **14**, 4998–5008 (2021). 10.1039/D1EE01495A

[11] Y. Shi, Y. Ji, J. Long, Y. Liang, Y. Liu et al., Unveiling hydrocerussite as an electrochemically stable active phase for efficient carbon dioxide electroreduction to formate. Nat. Commun. **11**, 3415 (2020). 10.1038/s41467-020-17120-9 32641692 10.1038/s41467-020-17120-9 PMC7343827

[12] H. Shang, T. Wang, J. Pei, Z. Jiang, D. Zhou et al., Design of a single-atom indium^δ+^–N_4_ interface for efficient electroreduction of CO_2_ to formate. Angew. Chem. Int. Ed. **59**, 22465–22469 (2020). 10.1002/anie.202010903 10.1002/anie.202010903 32876989

[13] L. Li, A. Ozden, S. Guo, A.D.A.F.P. Garci, C. Wang et al., Stable active CO2 reduction to formate via redox-modulated stabilization of active sites. Nat. Commun. **12**, 5223 (2021). 10.1038/s41467-021-25573-9 34471135 10.1038/s41467-021-25573-9 PMC8410779

[14] W. Wang, Z. Wang, R. Yang, J. Duan, Y. Liu et al., In Situ phase separation into coupled interfaces for promoting CO_2_ electroreduction to formate over a wide potential window. Angew. Chem. Int. Ed. **60**, 22940–22947 (2021). 10.1002/anie.202110000 10.1002/anie.202110000 34387932

[15] Y. Chen, M.W. Kanan, Tin oxide dependence of the CO_2_ reduction efficiency on tin electrodes and enhanced activity for tin/tin oxide thin-film catalysts. J. Am. Chem. Soc. **134**, 1986–1989 (2012). 10.1021/ja2108799 22239243 10.1021/ja2108799

[16] W. Luc, C. Collins, S. Wang, H. Xin, K. He et al., Ag–Sn bimetallic catalyst with a core-shell structure for CO_2_ reduction. J. Am. Chem. Soc. **139**, 1885–1893 (2017). 10.1021/jacs.6b10435 28094994 10.1021/jacs.6b10435

[17] K. Ye, Z. Zhou, J. Shao, L. Lin, D. Gao et al., In situ reconstruction of a hierarchical Sn–Cu/SnO_x_ core/shell catalyst for high-performance CO_2_ electroreduction. Angew. Chem. Int. Ed. **59**, 4814–4821 (2020). 10.1002/anie.201916538 10.1002/anie.201916538 31944516

[18] H. Liu, B. Li, Z. Liu, Z. Liang, H. Chuai et al., Ceria-mediated dynamic Sn^0^/Sn^δ+^ redox cycle for CO_2_ electroreduction. ACS Catal. **13**, 5033–5042 (2023). 10.1021/acscatal.2c06135

[19] Y. Jiang, J. Shan, P. Wang, L. Huang, Y. Zheng et al., Stabilizing oxidation state of SnO_2_ for highly selective CO_2_ electroreduction to formate at large current densities. ACS Catal. **13**, 3101–3108 (2023). 10.1021/acscatal.3c00123

[20] M. Chen, S. Wan, L. Zhong, D. Liu, H. Yang et al., Dynamic restructuring of Cu-Doped SnS_2_ nanoflowers for highly selective electrochemical CO_2_ reduction to formate. Angew. Chem. Int. Ed. **60**, 26233–26237 (2021). 10.1002/ange.202111905 10.1002/anie.202111905 34586693

[21] C. Chai, B. Liu, K. Liu, P. Li, J. Fu et al., Heteroatoms induce localization of the electric field and promote a wide potential-window selectivity towards CO in the CO_2_ electroreduction. Angew. Chem. Int. Ed. **61**, e202212640 (2022). 10.1002/anie.202212640 10.1002/anie.202212640 PMC9828093 36074055

[22] T. Wang, J. Chen, X. Ren, J. Zhang, J. Ding et al., Halogen-incorporated Sn catalysts for selective electrochemical CO_2_ reduction to formate. Angew. Chem. Int. Ed. **62**, e202211174 (2023). 10.1002/anie.202211174 10.1002/anie.202211174 36562773

[23] S. Yan, C. Peng, C. Yang, Y. Chen, J. Zhang et al., Electron localization and lattice strain induced by surface lithium doping enable ampere-level electrosynthesis of formate from CO_2_. Angew. Chem. Int. Ed. **60**, 25741–25745 (2021). 10.1002/ange.202111351 10.1002/anie.202111351 34617366

[24] M. Yu, G.H. Moon, R.G. Castillo, S. DeBeer, C. Weidenthaler et al., Dual role of silver moieties coupled with ordered mesoporous cobalt oxide towards electrocatalytic oxygen evolution reaction. Angew. Chem. Int. Ed. **59**, 16544–16552 (2020). 10.1002/ange.202003801 10.1002/anie.202003801 PMC7540465 32537829

[25] Q. Chen, K. Liu, Y. Zhou, X. Wang, K. Wu et al., Ordered Ag nanoneedle arrays with enhanced electrocatalytic CO_2_ reduction via structure-induced inhibition of hydrogen evolution. Nano Lett. **22**, 6276–6284 (2022). 10.1021/acs.nanolett.2c01853 35913397 10.1021/acs.nanolett.2c01853

[26] S.A. Chala, M.C. Tsai, W.N. Su, K.B. Ibrahim, B. Thirumalraj et al., Hierarchical 3D architectured Ag nanowires shelled with NiMn-layered double hydroxide as an efficient bifunctional oxygen electrocatalyst. ACS Nano **14**, 1770–1782 (2020). 10.1021/acsnano.9b07487 32003975 10.1021/acsnano.9b07487

[27] Z. Zhang, X. Li, C. Zhong, N. Zhao, Y. Deng et al., Spontaneous synthesis of silver-nanoparticle-decorated transition-metal hydroxides for enhanced oxygen evolution reaction. Angew. Chem. Int. Ed. **59**, 7245–7250 (2020). 10.1002/anie.202001703 10.1002/anie.202001703 32077180

[28] R. Gao, Z. Yang, L. Zheng, L. Gu, L. Liu et al., Enhancing the catalytic activity of Co_3_O_4_ for Li–O_2_ batteries through the synergy of surface/interface/doping engineering. ACS Catal. **8**, 1955–1963 (2018). 10.1021/acscatal.7b03566

[29] H. Wu, F. Huang, J. Peng, Y. Cao, High-efficiency electron injection cathode of Au for polymer light-emitting devices. Org. Electron. **6**, 118–128 (2005). 10.1016/j.orgel.2005.03.009

[30] A.W. Dweydari, C.H.B. Mee, Work function measurements on (100) and (110) surfaces of silver. Phys. Status Solidi **27**, 223–230 (1975). 10.1002/pssa.2210270126

[31] Z. Yu, Z. Yang, Z. Ni, Y. Shao, B. Chen et al., Simplified interconnection structure based on C_60_/SnO_2-x_ for all-perovskite tandem solar cells. Nat. Energy **5**, 657–665 (2020). 10.1038/s41560-020-0657-y

[32] H. Yang, Q. Lin, C. Zhang, X. Yu, Z. Cheng et al., Carbon dioxide electroreduction on single-atom nickel decorated carbon membranes with industry compatible current densities. Nat. Commun. **11**, 593 (2020). 10.1038/s41467-020-14402-0 32001699 10.1038/s41467-020-14402-0 PMC6992760

[33] S. Liu, X.F. Lu, J. Xiao, X. Wang, X.W.D. Lou, Bi_2_O_3_ nanosheets grown on multi-channel carbon matrix to catalyze efficient CO_2_ electroreduction to HCOOH. Angew. Chem. Int. Ed. **58**, 13828–13833 (2019). 10.1002/ange.201907674 10.1002/anie.201907674 31347752

[34] P. Zhu, H. Wang, High-purity and high-concentration liquid fuels through CO_2_ electroreduction. Nat. Catal. **4**, 943–951 (2021). 10.1038/s41929-021-00694-y

[35] A. Manthiram, X. Yu, S. Wang, Lithium battery chemistries enabled by solid-state electrolytes. Nat. Rev. Mater. **2**, 16103 (2017). 10.1038/natrevmats.2016.103

[36] L. Fan, S. Wei, S. Li, Q. Li, Y. Lu, Recent progress of the solid-state electrolytes for high-energy metal-based batteries. Adv. Energy Mater. **8**, 1702657 (2018). 10.1002/aenm.201702657

[37] N. Han, Y. Wang, J. Deng, J. Zhou, Y. Wu et al., Self-templated synthesis of hierarchical mesoporous SnO_2_ nanosheets for selective CO_2_ reduction. J. Mater. Chem. A **7**, 1267–1272 (2019). 10.1039/C8TA10959A

[38] G. Kresse, J. Furthmüller, J. Ab initio molecular dynamics for liquid metals. Comp. Mater. Sci. **6**, 15–50 (1996). 10.1103/PhysRevB.47.558 10.1103/physrevb.47.558 10004490

[39] G. Kresse, J. Hafner, Ab initio molecular dynamics for liquid metals. Phys. Rev. B **47**, 558 (1993). 10.1103/PhysRevB.47.558 10.1103/physrevb.47.558 10004490

[40] P.E. Blöchl, Projector augmented-wave method. Phys. Rev. B **50**, 17953 (1994). 10.1103/PhysRevB.50.17953 10.1103/physrevb.50.17953 9976227

[41] J.P. Perdew, K. Burke, M. Ernzerhof, Generalized gradient approximation made simple. Phys. Rev. Lett. **77**, 3865 (1996). 10.1103/PhysRevLett.77.3865 10062328 10.1103/PhysRevLett.77.3865

[42] L. Zhang, W. Cai, N. Bao, H. Yang, Implanting an electron donor to enlarge the d-p hybridization of high-entropy (oxy)hydroxide: a novel design to boost oxygen evolution. Adv. Mater. **34**, e2110511 (2022). 10.1002/adma.202110511 35259283 10.1002/adma.202110511

[43] P. Wang, M. Qiao, Q. Shao, Y. Pi, X. Zhu et al., Phase and structure engineering of copper tin heterostructures for efficient electrochemical carbon dioxide reduction. Nat. Commun. **9**, 4933 (2018). 10.1038/s41467-018-07419-z 30467320 10.1038/s41467-018-07419-z PMC6250663

[44] Q. Wang, K. Liu, K. Hu, C. Cai, H. Li et al., Attenuating metal-substrate conjugation in atomically dispersed nickel catalysts for electroreduction of CO_2_ to CO. Nat. Commun. **13**, 6082 (2022). 10.1038/s41467-022-33692-0 36241631 10.1038/s41467-022-33692-0 PMC9568552

[45] Y. Wang, C. Wang, Y. Wei, F. Wei, L. Kong et al., Efficient and selective electroreduction of CO_2_ to HCOOH over Bismuth-based bromide perovskites in acidic electrolytes. Chem. Eur. J. **28**, e202201832 (2022). 10.1002/chem.202201832 35853829 10.1002/chem.202201832

[46] J. Hao, Z. Zhuang, J. Hao, K. Cao, Y. Hu et al., Strain relaxation in metal alloy catalysts steers the product selectivity of electrocatalytic CO_2_ reduction. ACS Nano **16**, 3251–3263 (2022). 10.1021/acsnano.1c11145 35089016 10.1021/acsnano.1c11145

[47] L. Fan, C. Xia, P. Zhu, Y. Lu, H. Wang, Electrochemical CO_2_ reduction to high-concentration pure formic acid solutions in an all-solid-state reactor. Nat. Commun. **11**, 3633 (2020). 10.1038/s41467-020-17403-1 32686669 10.1038/s41467-020-17403-1 PMC7371694

