# Supplementary material for: Strongly Coupled Ag/Sn–SnO2 Nanosheets Toward CO2 Electroreduction to Pure HCOOH Solutions at Ampere-Level Current
Source: Nanomicro Lett. 2023 Dec 13;16:50. doi: 10.1007/s40820-023-01264-6 (PMC10719210; doi:10.1007/s40820-023-01264-6)
Supplement: Supplementary file 1 — Supplementary file1 (PDF 3577 KB) [file 40820_2023_1264_MOESM1_ESM.pdf]

Nano-Micro Letters

Supporting Information for

Strongly Coupled Ag/Sn-SnO_2 Nanosheets toward CO_2 Electroreduction to Pure HCOOH Solutions at Ampere-Level Current

Min Zhang[1], Aihui Cao[2], Yucui Xiang[1], Chaogang Ban[1], Guang Han[3, 4, *], Junjie Ding[1], Li-Yong Gan[1, 4, 5, *], and Xiaoyuan Zhou[1, 4, 5, *]

[1] College of Physics and Center of Quantum Materials and Devices, Chongqing University, Chongqing 401331, P. R. China

[2] State Key Laboratory of Structural Chemistry, Fujian Institute of Research on the Structure of Matter, Chinese Academy of Sciences (CAS), Fuzhou 350002, P. R. China

[3] College of Materials Science and Engineering, Chongqing University, Chongqing 400044, P. R. China

[4] Institute of New Energy Storage Materials and Equipment, Chongqing 401135, P. R. China

[5] State Key Laboratory of Coal Mine Disaster Dynamics and Control, Chongqing University, Chongqing 400044, P. R. China

*Corresponding authors. E-mail: xiaoyuan2013@cqu.edu.cn (Xiaoyuan Zhou); ganly@cqu.edu.cn (Li-Yong Gan), guang.han@cqu.edu.cn (Guang Han)

S1 Experimental Procedures

S1.1 Characterization

Powder X-ray diffraction (PXRD) patterns were recorded on an Empyrean X-ray diffractometer at 40 kV and 40 mA with Cu Kα radiation in 2θ ranging from 10° to 80°. Field emission scanning electron microscopy (FESEM) was performed on a Quattro S microscope operated at 5 kV. Transmission electronic microscopy (TEM) and high-resolution TEM (HRTEM) equipped with energy dispersive X-ray spectrometer (EDX) were conducted on a Talos-F200S microscope operated at 200 kV. X-ray photoelectron spectroscopy (XPS) and Ultroviolet Photoelectron Spectrometer (UPS) analyses were performed on a Thermo Fisher Scientific XPS ESCALAB 250Xi instrument with an Al-Ka (1486.8 eV) X-ray source. The X-ray absorption spectra of Ag and Sn K-edge (XANES and EXAFS experiments) were carried out in fluorescence mode at the BL14W1 beamline of the Shanghai Synchrotron Radiation Facility (SSRF), China. The EXAFS raw data were then background-subtracted, normalized and Fourier transformed by the standard procedures with the IFEFFIT package. Operando attenuated total reflection-infrared (ATR-IR) spectra were recorded on the IRTracer100 instrument. 1H NMR was conducted on AVANCE NEO 400 evolution with DMSO as internal standard.

S1.2 Electrochemical Measurements

During the electrolysis, gaseous products were detected by an on-line gas chromatography (GC) (Agilent 7820A) equipped with a molecular sieve 5 Å and two porapak Q columns continuously. The concentration of H_2 and CO was analyzed by a thermal conductivity detector (TCD) and a flame ionization detector (FID), respectively. The quantification of gaseous products was carried out using a conversion factor derived from the standard calibration gases. Liquid products were collected at the end of each electrocatalysis and analyzed by ^{1}H NMR (Aligent

DD2-400). For the NMR, 0.5 mL of the catholyte was mixed with 0.1 mL of D_2O containing dimethyl sulfoxide (DMSO, 10 ppm) as the internal standard. The concentration of formate was quantitatively determined from its NMR peak area relative to that of the internal standard using the calibration curve from a series of standard formate solutions.

The half-cell cathodic energy efficiency (CEE) was calculated as follows:

$$CEE=(1.23–E_{formate})/(1.23–E_{applied})\times FE_{formate}\times 100\%$$

where $E_{formate}$ = –0.2 V vs. RHE is the thermodynamic potential of CO_2 reduction to formate. $FE_{formate}$ is the Faradaic efficiency of formate production. $E_{applied}$ is the applied potential vs. RHE.

The electrochemical impedance spectroscopy (EIS) was recorded at –0.73 V with the frequency ranging from 0.1 to 10^5 Hz at the AC amplitude of 5 mV.

The adsorption of $OH^–$ as a surrogate of $CO_2{}^{*–}$ was examined through a cathodic LSV scan between –0.4 and 0.38 V in Ar-saturated aqueous 0.1 M KOH solution at a scan rate of 10 mV $s^{–1}$.

Electrochemically active surface area (ECSA) was obtained by measuring the electrochemical double-layer capacitance from the scan-rate dependence of CVs in a potential range of –0.18 to –0.08 V. The electrochemical impedance spectroscopy (EIS) was recorded at –0.73 V with the frequency ranging from 0.1 to 10^5 Hz at the AC amplitude of 5 mV.

For ATR-IR measurements, the loaded Ag/Sn-SnO_2 (or Sn-SnO_2) electrode was used as the working electrode, along with Ag/AgCl as the reference electrode, and platinum wire as the counter electrode.

S2 Supplementary Figures and Tables

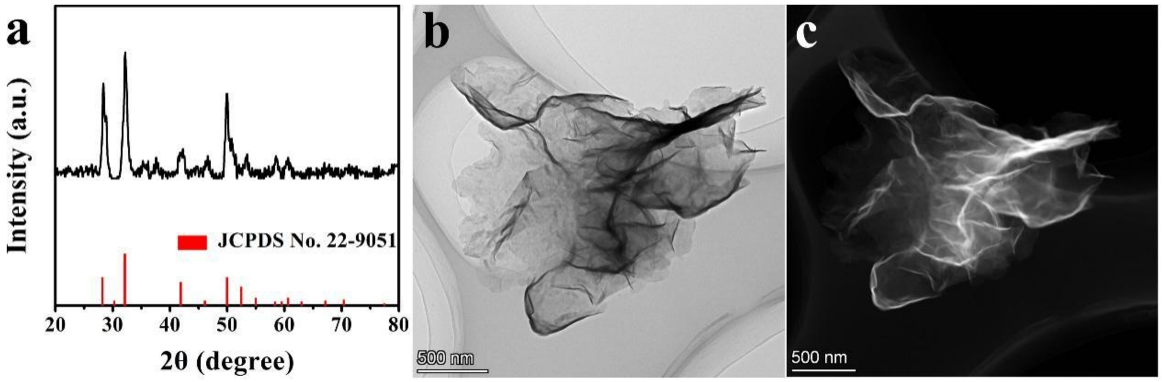

Fig. S1 Characterization of SnS_2 NSs: (**a**) XRD pattern; (**b**) TEM image; (**c**) HAADF-STEM image

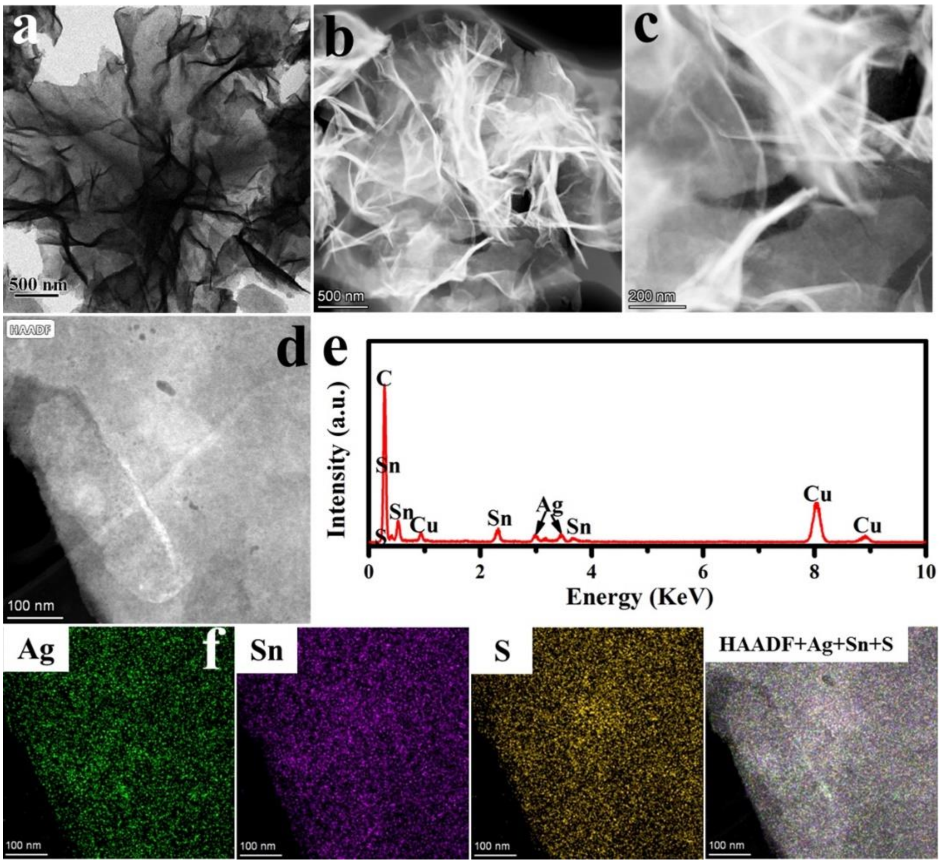

Fig. S2 Characterization of Ag-SnS_2 NSs: (**a**) TEM image; (**b, c**) STEM images; (**d**) HAADF-STEM image; (**e**) corresponding EDX spectrum; (**f**) EDX mapping images

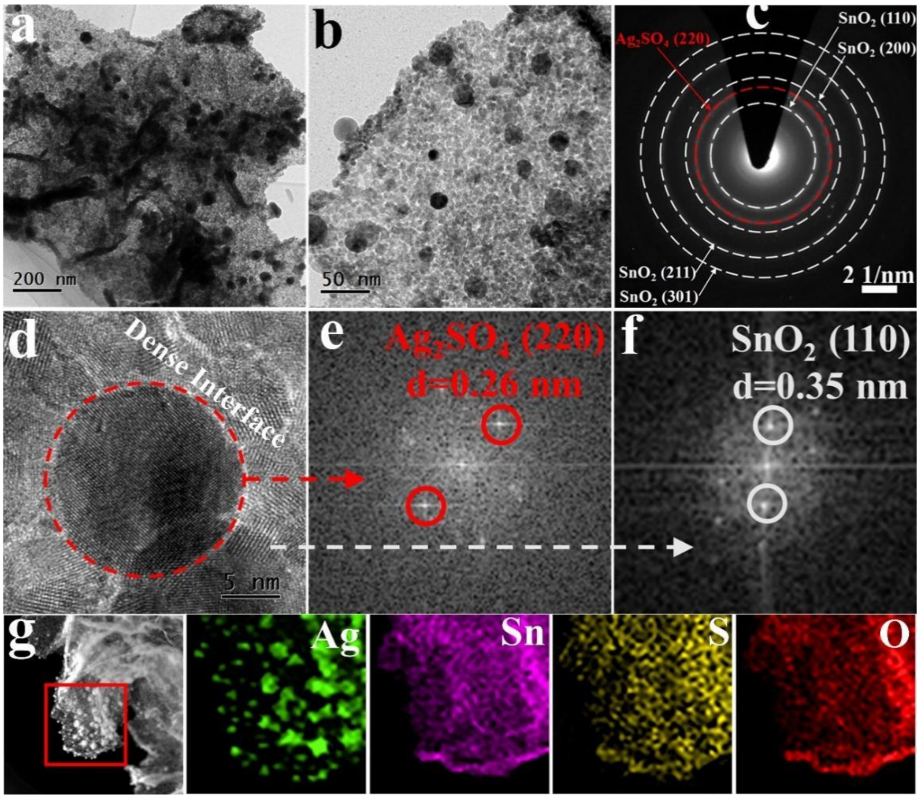

Fig. S3 Characterization of Ag_2SO_4/SnO_2 NSs: (**a, b**) TEM images; (**c**) SAED pattern; (**d**) HRTEM image; (**e, f**) corresponding FFT patterns; (**g**) HAADF-STEM and EDX mapping images

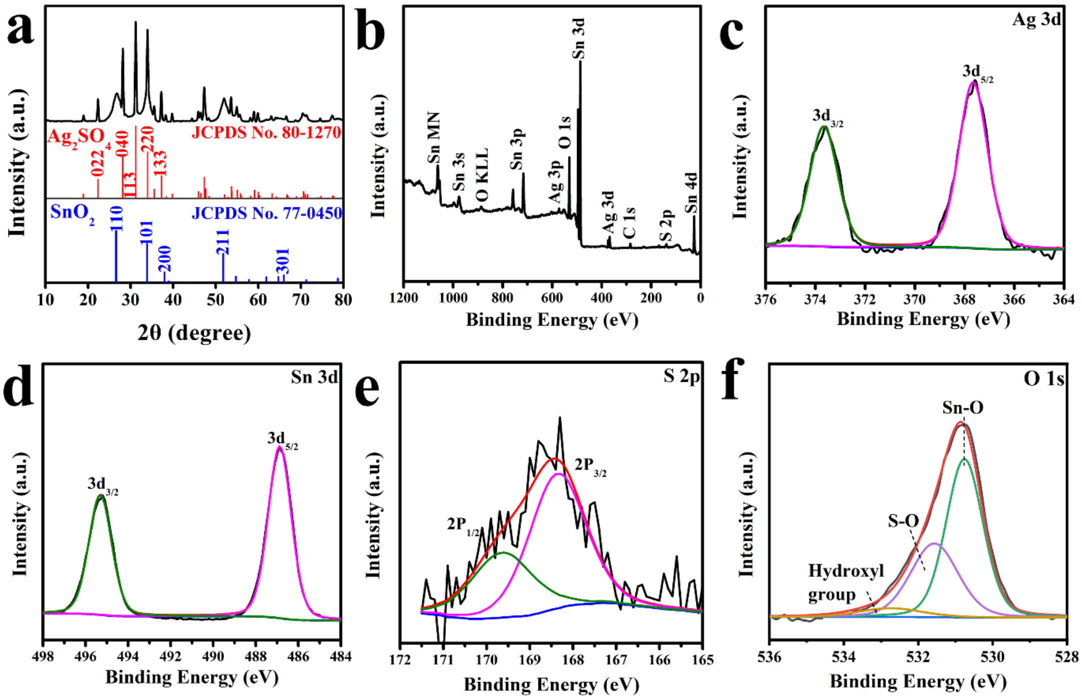

Fig. S4 Characterization of Ag_2SO_4/SnO_2 NSs: (**a**) PXRD pattern; XPS spectra of (**b**) full spectrum, (**c**) Ag 3d spectrum, (**d**) Sn 3d spectrum, (**e**) S 2p spectrum, (**f**) O 1s spectrum.

PXRD pattern exhibits that all the diffraction peaks of the product can be indexed to the Ag_2SO_4 and SnO_2 phase (**Fig. S4a**). The surface chemical state and composition of the Ag_2SO_4/SnO_2 NSs were further characterized by X-ray photoelectron spectroscopy (XPS). As shown in **Fig. S4b**, there are a series of peaks corresponding to Sn, Ag, O, C, and S elements in the survey spectrum of the Ag_2SO_4/SnO_2 NSs. Ag 3d spectra showed Ag $3d_{5/2}$ and Ag $3d_{3/2}$ peaks at binding energies of 367.5 and 373.5 eV, respectively, indicating the metallic Ag^+ characteristics of sample surfaces (**Fig. S4c**). **Fig.** S4d shows the Sn 3d spectrum, in which peaks at 494.6 and 486.3 eV correspond to Sn $3d_{3/2}$ and $3d_{5/2}$ orbitals of Sn^{4+}. In the S 2p spectra (**Fig. S4e**), two splitting peaks around 168.6 and 169.8 eV are assigned to S^{6+} $2p_{3/2}$ and $2p_{1/2}$ orbitals. In the O 1s spectrum (**Fig. S4f**), the peak at 531.9 eV is associating with the S-O bonds in SO_4^{2-} species, and the peak at 529.9 eV is assigned to the Sn-O bonds. In addition, a peak around 533 eV was also detected which is ascribed to hydroxyl groups.

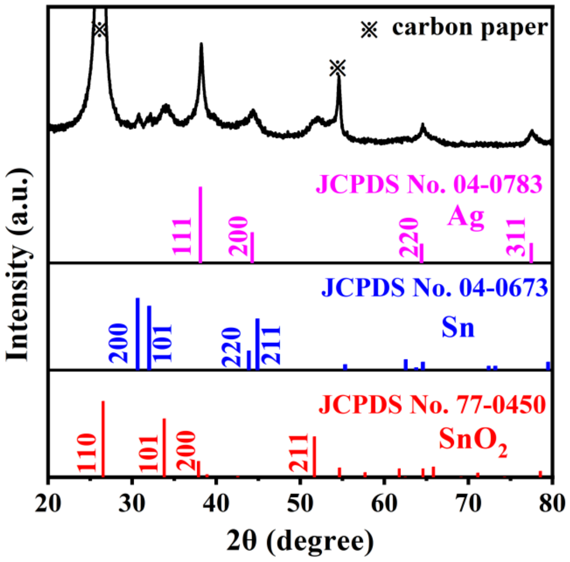

Fig. S5 XRD pattern of $Ag/Sn\text{-}SnO_2$ NSs

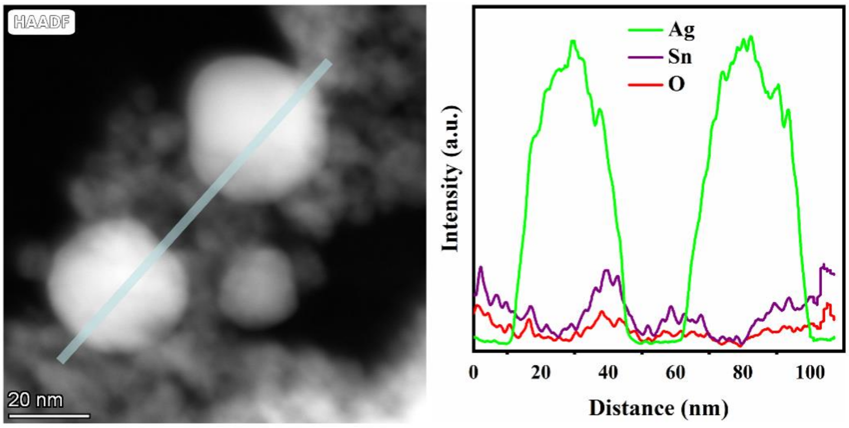

Fig. S6 HAADF-STEM image and corresponding EDX line scans of Ag/Sn-SnO_2 NSs

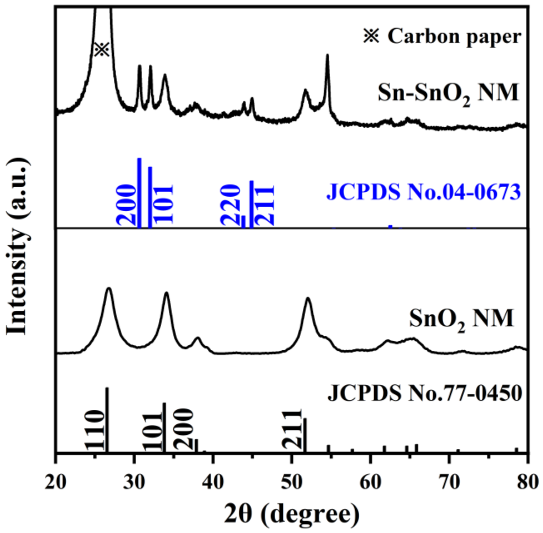

Fig. S7 XRD patterns of SnO_2 NSs and Sn-SnO_2 NSs

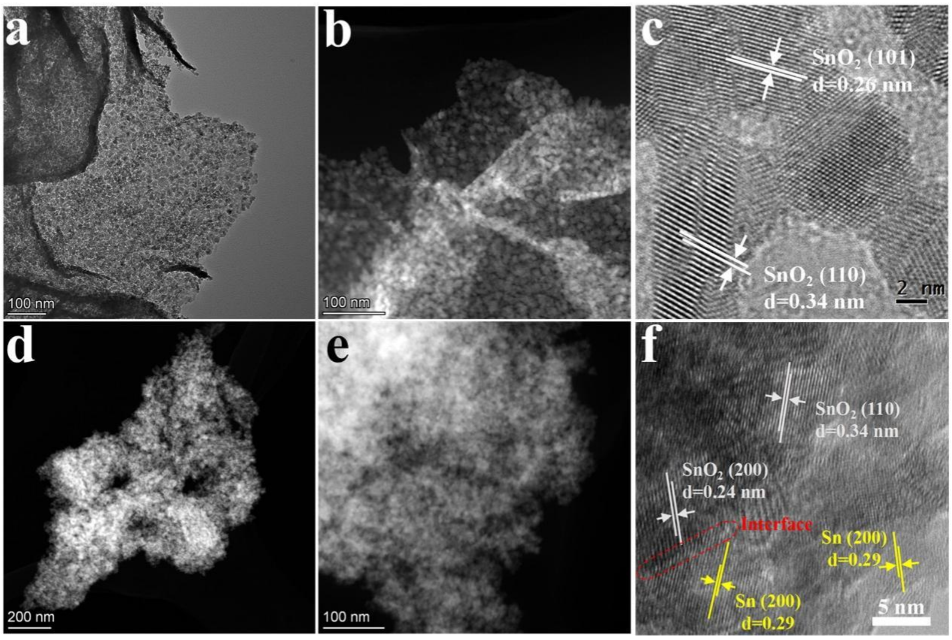

Fig. S8 Characterization of SnO_2 NSs and derived Sn-SnO_2 NSs: (**a**) TEM image, (**b**) HAADF-STEM image, (**c**) HRTEM image of SnO_2 NSs; (**d, e**) HAADF-STEM images, (**f**) HRTEM image of Sn-SnO_2 NSs

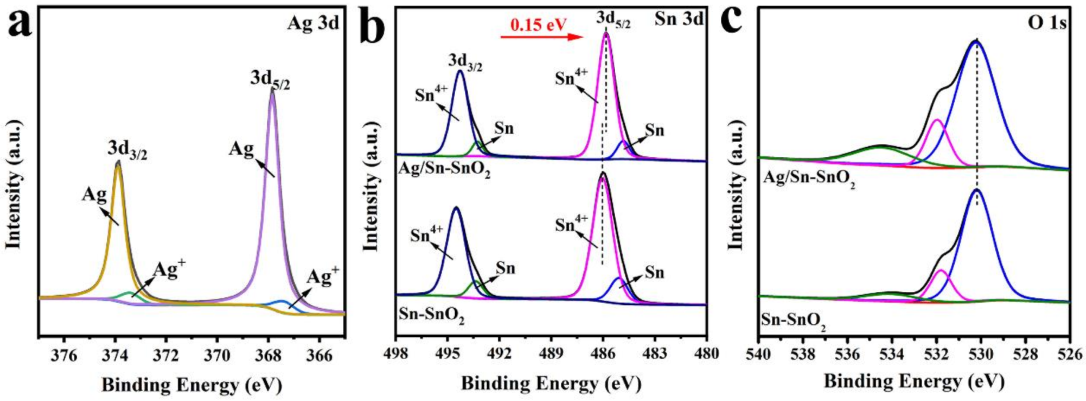

Fig. S9 High-resolution XPS spectra: (**a**) Ag 3d of Ag/Sn-SnO_2 NSs; (**b**) Sn 3d of Ag/Sn-SnO_2 NSs and Sn-SnO_2 NSs; (**c**) O 1s of Ag/Sn-SnO_2 NSs and Sn-SnO_2 NSs

The O 1s spectra for Ag/Sn-SnO_2 NSs and Sn-SnO_2 NSs were resolved into three peaks at the binding energies of 529.4, 531.6, and 534.2 eV, corresponding to the lattice oxygen of Sn–O, oxygen atoms adjacent to O_{vacs}, and the adsorbed oxygen (O_{ads}), respectively (**Fig. S9c**).

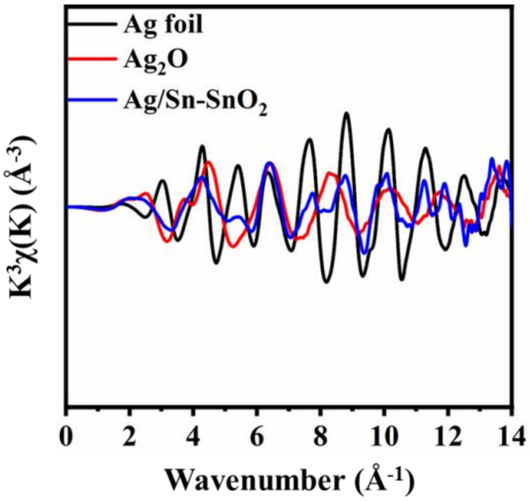

Fig. S10 Ag K-edge EXAFS oscillations of Ag foil, Ag_2O and Ag/Sn-SnO_2

The oscillation curves of Ag K-edge for Ag/Sn-SnO_2 in the K range of 0–14.0 $Å^{-1}$ also displayed obvious decrease in oscillation intensity with respect to Ag foil and Ag_2O (**Fig. S10**), indicating the existence of two silver phases.

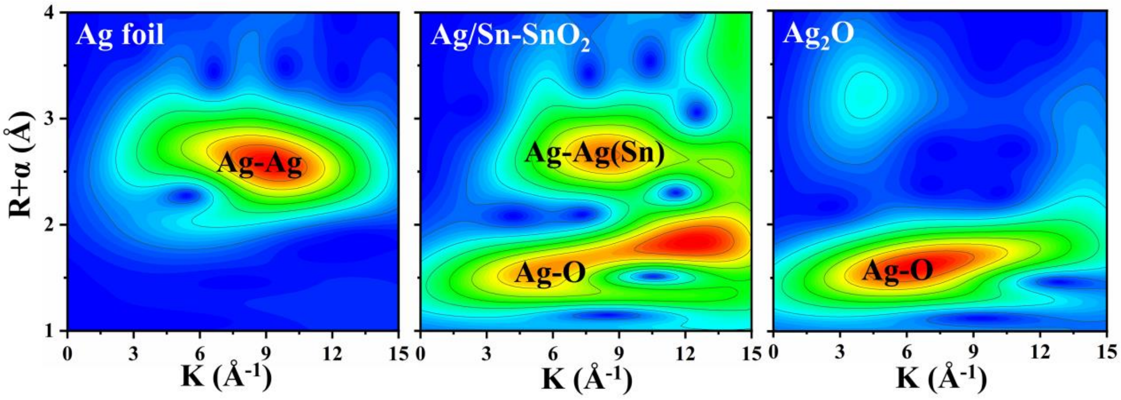

Fig. S11 Wavelet transform EXAFS of Ag of Ag foil, Ag/Sn-SnO_2, and Ag_2O

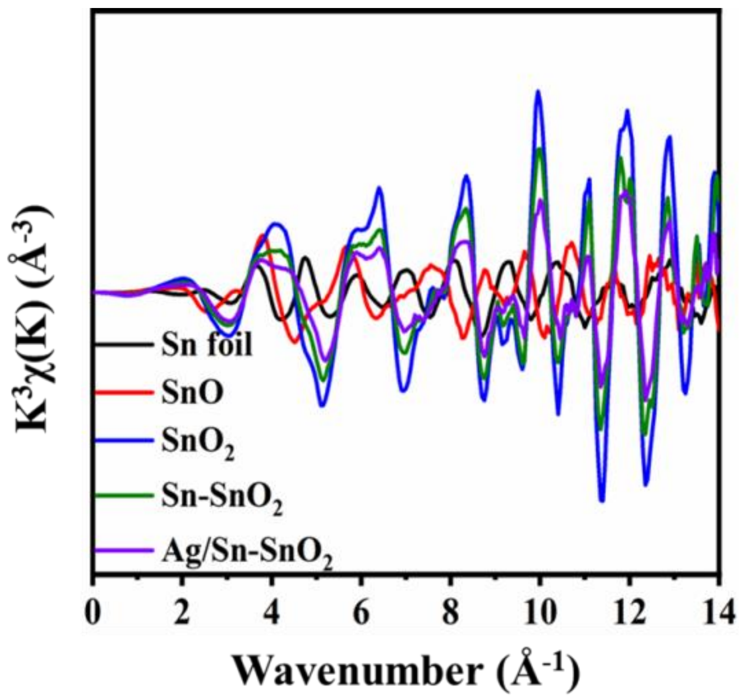

Fig. S12 Sn K-edge EXAFS oscillations of Sn foil, SnO_2, Sn-SnO_2, Ag/Sn-SnO_2 and SnO

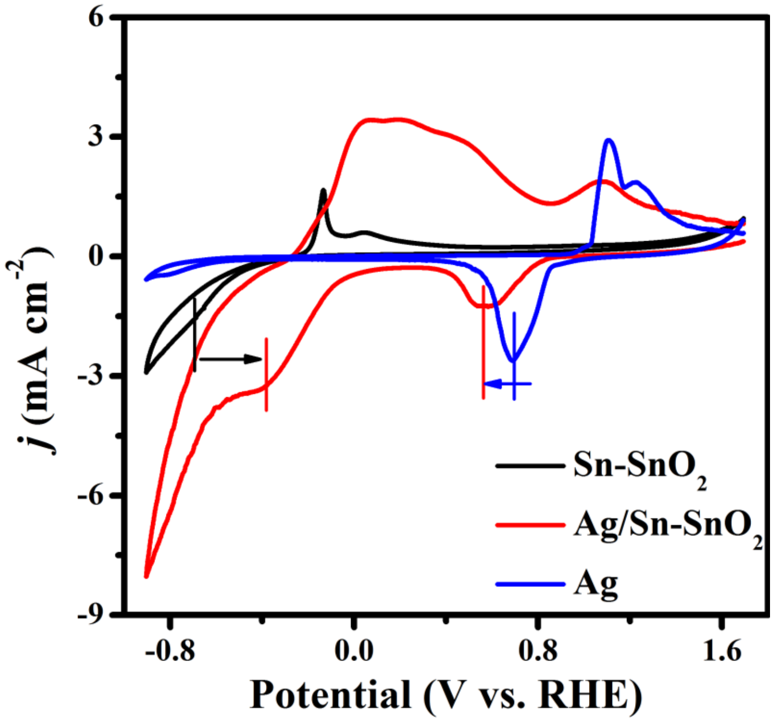

Fig. S13 The Sn^{4+}/Sn and Ag^{+}/Ag reduction waves of Ag/Sn-SnO_2 in cyclic voltammetry curves

The cyclic voltammetry (CV) test was first conducted in an Ar-saturated 0.5 M $KHCO_3$ electrolyte to investigate the Ag and Sn-SnO_2 interaction. As shown in **Fig. S13**, the Sn^{4+} to Sn reduction peak shifted from −0.39 V (for Sn-SnO_2) to −0.37 V (for Ag/Sn-SnO_2), while the Ag^{+}/Ag reduction peak shifted from 0.75 V (for Ag powder) to 0.63 V (for Ag/Sn-SnO_2), suggesting that the interphase electron transfer from Ag to Sn atoms, during which Sn atoms as electron acceptor became electron enrichment.

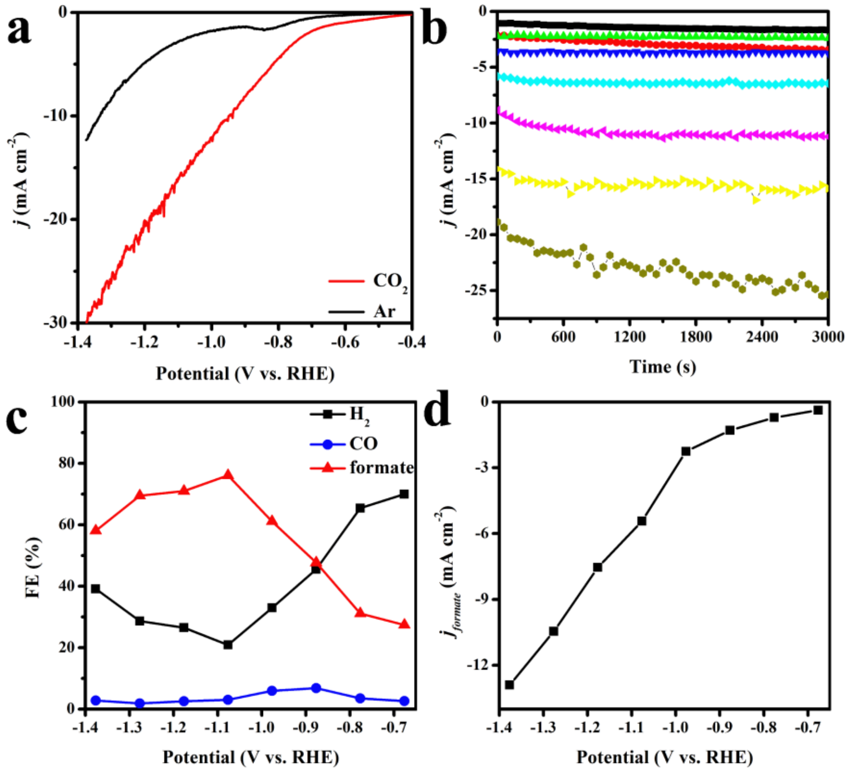

Fig. S14 Electrocatalytic CO_2RR performance of Sn-SnO_2 NSs in CO_2-saturated 0.5 M $KHCO_3$: (**a**) Polarization curves in Ar- or CO_2-saturated solution; (**b**) Total current densities at designated potentials; (**c**) FE for formate, CO and H_2; (**d**) Partial current densities of formate

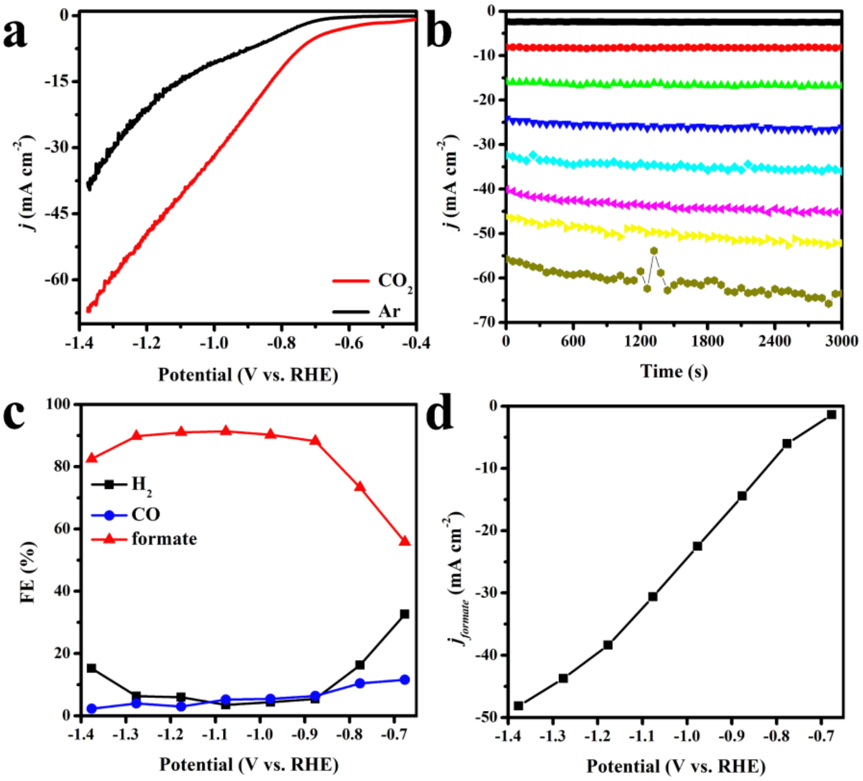

Fig. S15 Electrocatalytic CO_2RR performance of Ag/Sn-SnO_2 NSs in CO_2-saturated 0.5 M $KHCO_3$: (**a**) Polarization curves in Ar- or CO_2-saturated solution; (**b**) Total current densities at designated potentials; (**c**) FE for formate, CO and H_2; (**d**) Partial current densities of formate

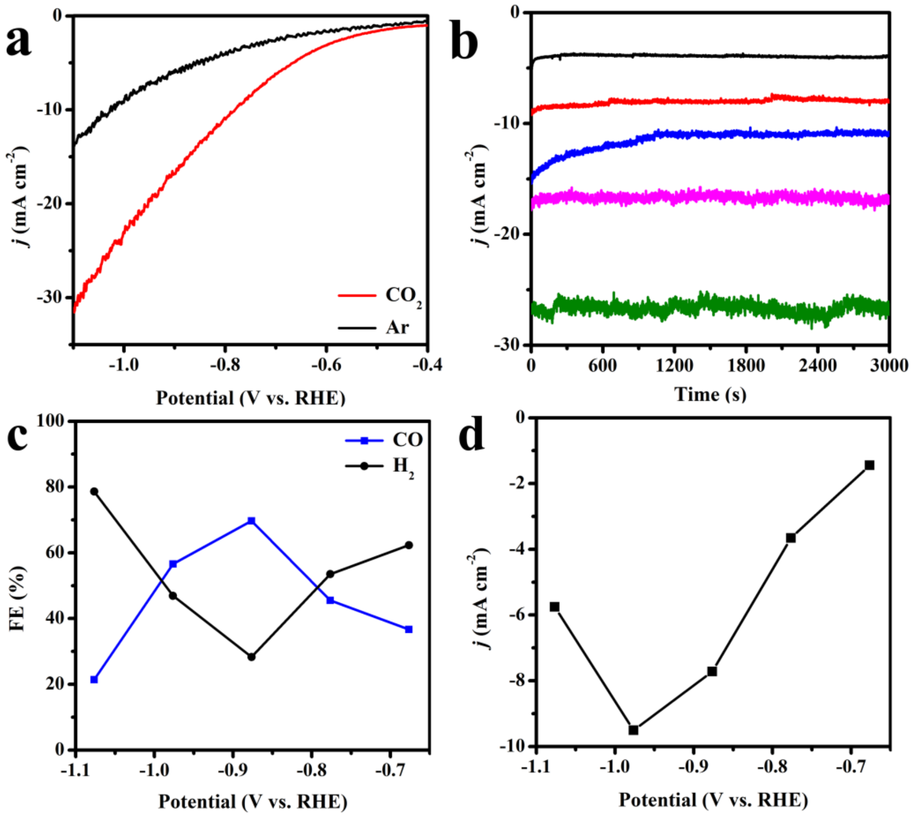

Fig. S16 Electrocatalytic CO_2RR performance of Ag powder in CO_2-saturated 0.5 M $KHCO_3$: (**a**) Polarization curves in Ar- or CO_2-saturated solution; (**b**) Total current densities at designated potentials; (**c**) FE for CO and H_2; (**d**) Partial current densities of CO

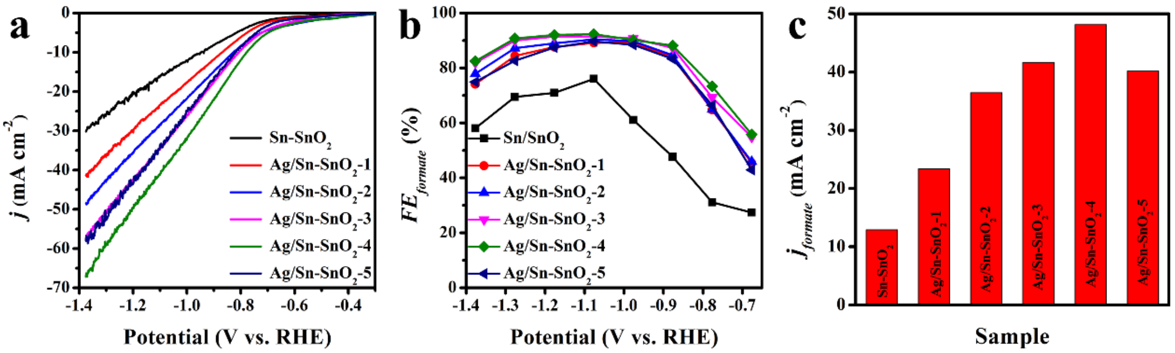

Fig. S17 Electrocatalytic CO_2RR performance of Ag powder in CO_2-saturated 0.5 M $KHCO_3$: (**a**) Polarization curves in CO_2-saturated solution; (**b**) FE for formate; (**c**) Partial current densities of formate

Where the content of Ag in Ag/Sn-SnO_2 was indirectly controlled by changing the concentration of Ag^+ in the ion exchange experiment. Firstly, 0.1 g of SnS_2 NSs were dispersed in 80 mL of ethanol with sonication for 30 min. Then, 20 mL of ethanol solution of $AgNO_3$ (0.12, 0.24, 0.36, 0.48, 0.5 mM, name 1, 2, 3, 4, 5) was added to the above mixture with magnetic stirring for 12 h at 60 °C. Subsequently, the Ag^+-exchanged SnS_2 NSs was collected, washed, and dried, and it was further calcined at 500 °C for 2 h in O_2 with a heating rate of 5 °C min^{-1}. Finally, Ag/Sn-SnO_2-X (X=1, 2, 3, 4, 5) were obtained via an in situ electrochemical self-reconstruction process.

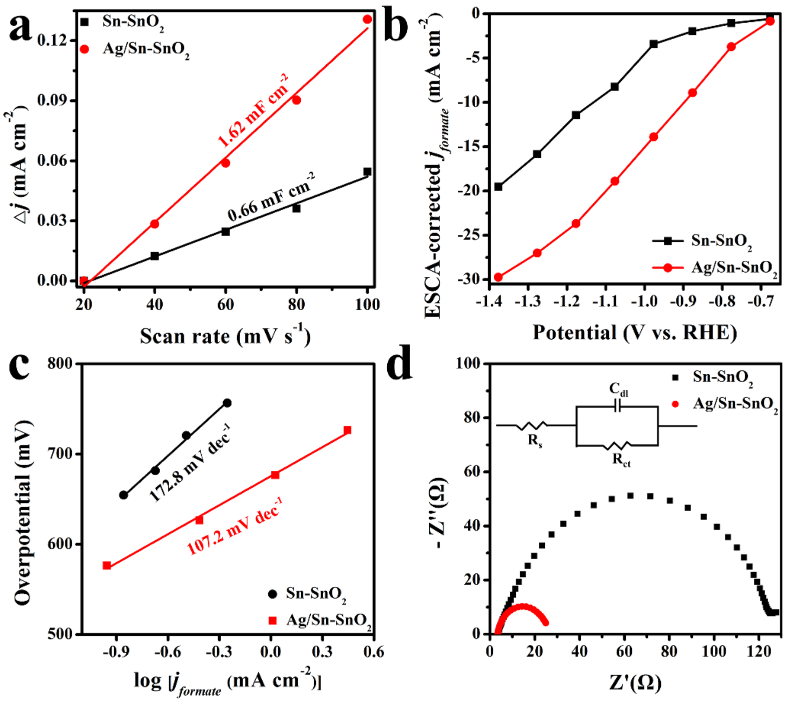

Fig. S18 The Sn-SnO_2 NSs and Ag/Sn-SnO_2 NSs for electrocatalytic CO_2 reduction: (**a**) ESCAs; (**b**) ESCAs-normalized formate partial current density; (**c**) Tafel plots; (**d**) Nyquist plots (insert is simplified randles circuit)

In a simplified Randles circuit, R_s and R_{ct} represent the solution resistance and charge transfer resistance, respectively. C_{dl} element models the double-layer capacitance. The kinetics for Faradaic CO_2RR is determined by charge transfer resistance (R_{ct}).

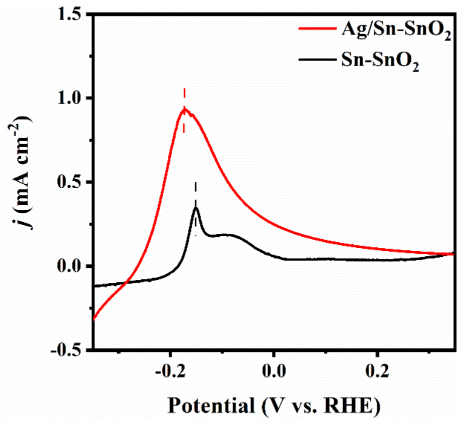

Fig. S19 LSV curves of Ag/Sn-SnO_2 NSs and Sn-SnO_2 NSs in Ar-saturated 0.1 M KOH electrolyte

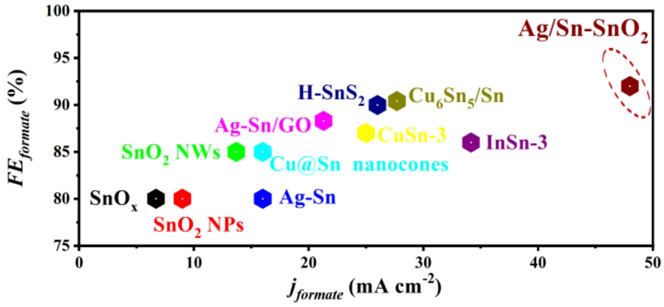

Fig. S20 Comparison of the performance of Ag/Sn-SnO_2 NSs with other Sn-based catalysts of H-type reactor

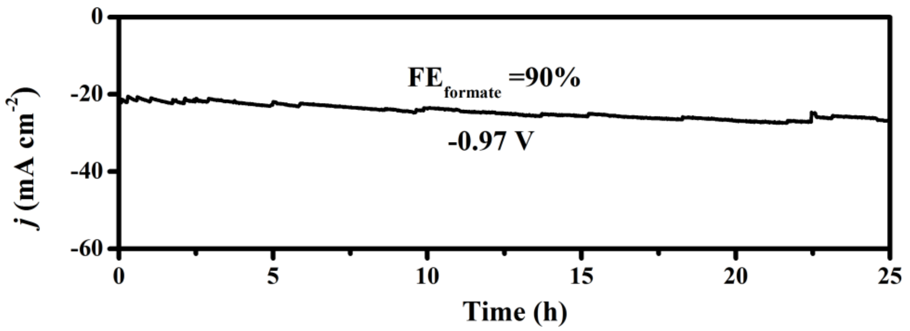

Fig. S21 Stability test for Ag/Sn-SnO_2 NSs at a potential of –0.97 V vs. RHE

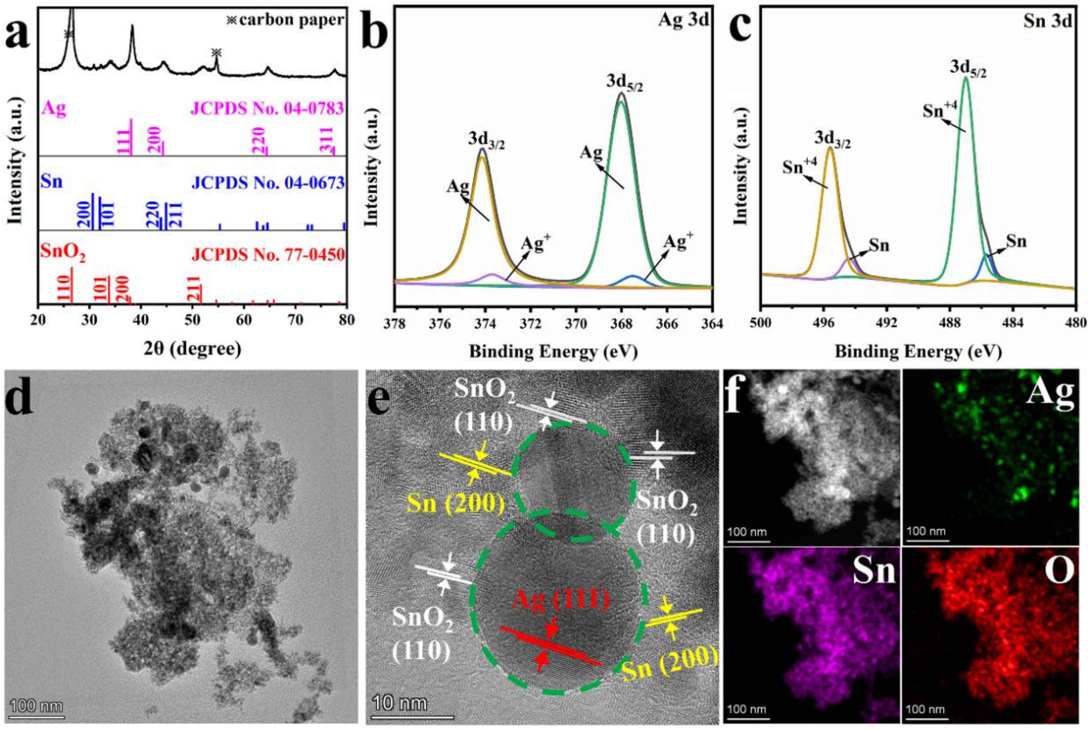

Fig. S22 The recycled Ag/Sn-SnO_2 NSs after stability test: (**a**) XRD pattern; high-resolution XPS spectra of (**b**) Ag 3d, and (**c**) Sn 3d; (**d**) TEM image; (**e**) HRTEM image; (**g**) corresponding EDS mapping images

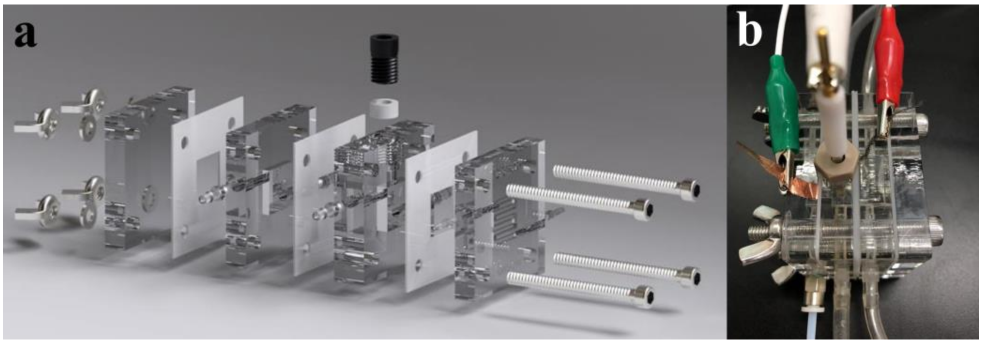

Fig. S23 Flow-type reactor configuration for electrocatalytic CO_2 reduction: (**a**) schematic diagram; (**b**) actual photograph

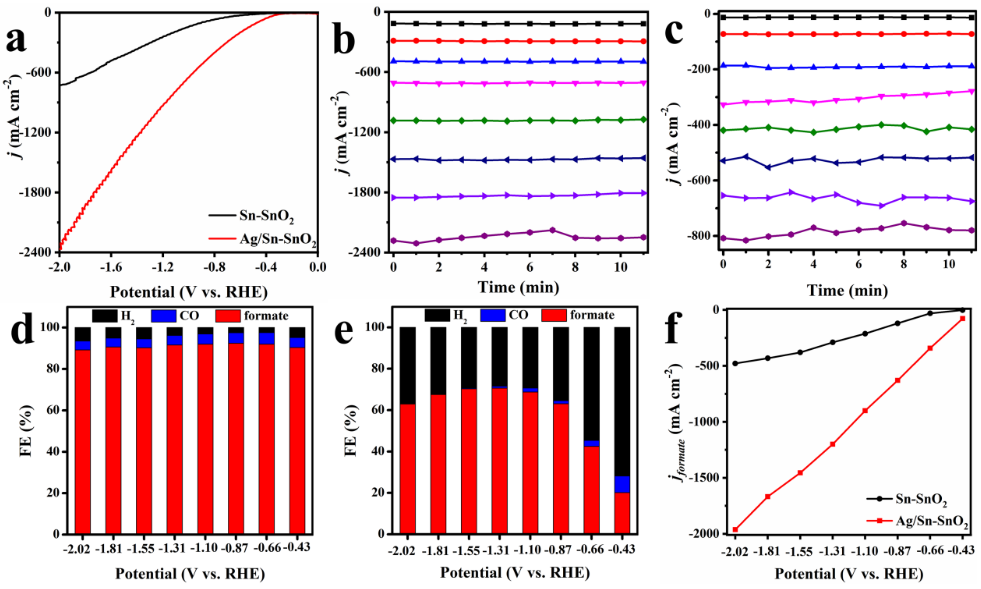

Fig. S24 Electrocatalytic CO_2RR performance in Flow-type reactor using 1M KOH as electrolyte: (**a**) Polarization curves; (**b, c**) Total current densities of Sn-SnO_2 NSs (**b**) and Ag/Sn-SnO_2 NSs (**c**); (**d, e**) FE for formate, CO and H_2 of Ag/Sn-SnO_2 NSs (**d**) and Sn-SnO_2 NSs (**e**); (**f**) Partial current densities of formate

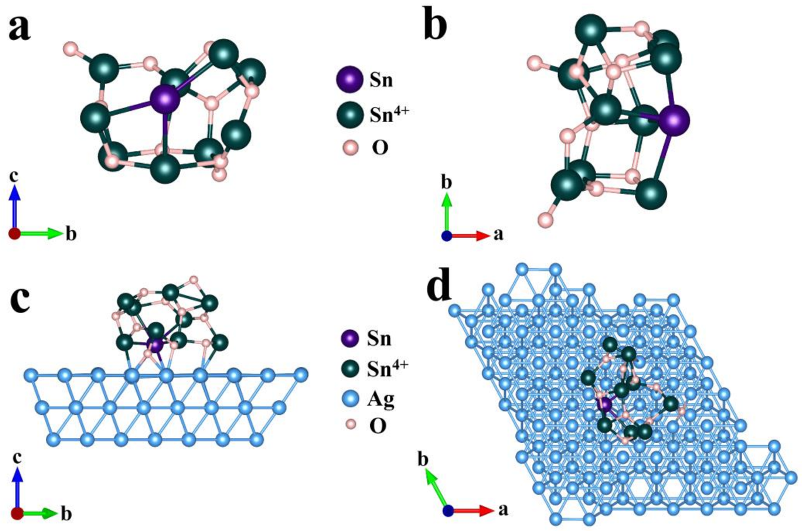

Fig. S25 Side- and top-view of the initial models: (**a, b**) Sn-SnO_2; (**c, d**) Ag/Sn-SnO_2

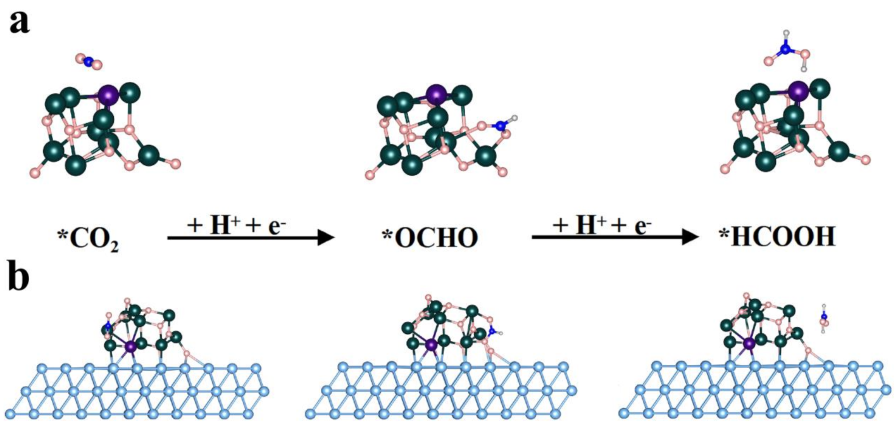

Fig. S26 The optimized intermediate species along the reaction pathways for CO_2 electroreduction to formate on the (**a**) Sn-SnO_2 and (**b**) Ag/Sn-SnO_2

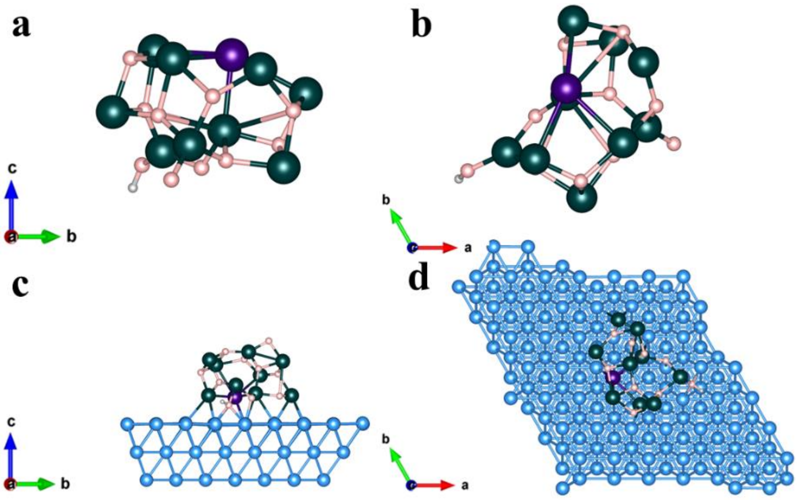

Fig. S27 Calculated adsorption configuration of *H on catalysts: (**a, b**) Sn-SnO_2; (**c, d**) Ag/Sn-SnO_2

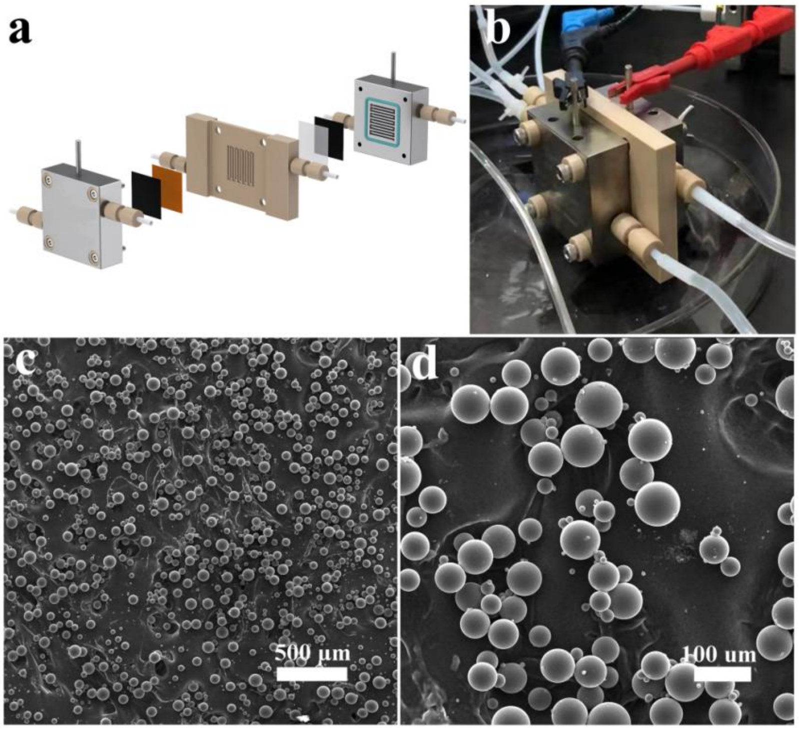

Fig. S28 MEA with PSE layer electrolyzer for pure HCOOH production: (**a, b**) Schematic diagram, and digital image; (**c, d**) SEM images of the sulfonated styrene-divinylbenzene copolymer proton conductor

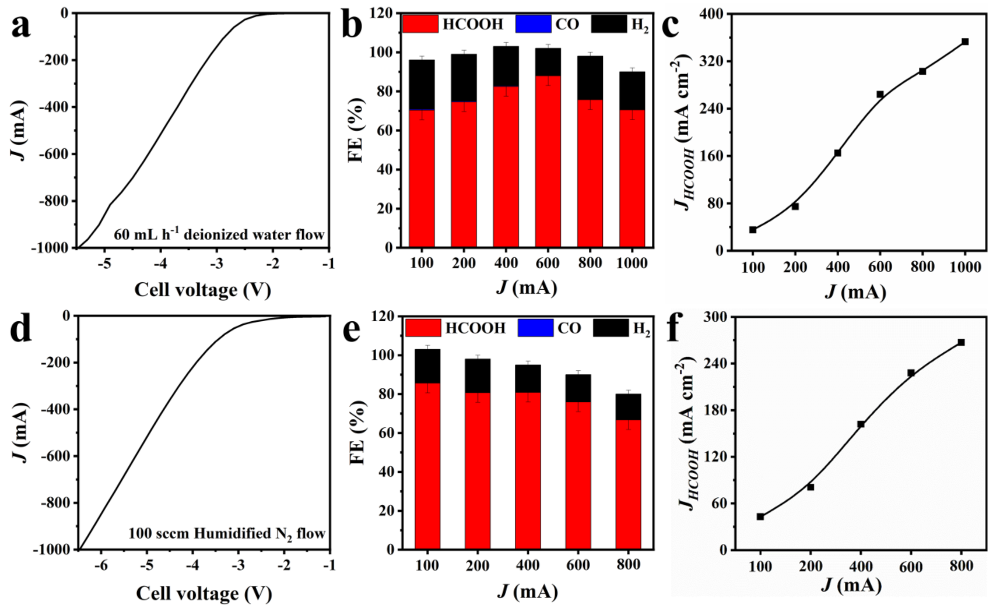

Fig. S29 Electrocatalytic CO_2RR performance of Ag/Sn-SnO_2 NSs in MEA with PSE layer reactor: (**a**) LSV curve, (**b**) FE of all products, (**c**) Partial current densities of HCOOH using DI water as carrier flow; (**d**) LSV curve, (**e**) FE of all products, (**f**) Partial current densities of HCOOH using humidified N_2 as carrier flow

Table S1 Ag K-edge EXAFS fitting results for Ag/Sn-SnO_2, Ag foil, and Ag_2O

sample	shell	N	σ^2 (Å^2)	ΔE_0 (eV)	R (Å)	R-factor
Ag foil	Ag-Ag	12	9.6±0.2	2.0±0.2	2.86±0.00	0.002
Ag_2O	Ag-O	4	1.2±1.9	2.0±1.5	2.05±0.01	0.020
Ag/Sn-SnO_2	Ag-O	1.1±0.2	5.4±5.9	0.9±1.3	2.03±0.01	0.013
	Ag-Ag	2.1±1.1		-8.1±4.1	2.83±0.03	
	Ag-Sn	2.3±1.2			2.92±0.02	

N: Coordination numbers; σ: Debye-Waller parameters, R: coordination distances; R-factor: goodness of fit, ΔE_0 energy shift. S_0^2 is amplitude reduction factor (S_0^2=0.83 was obtained by Ag foil fitting and applied for other sample fitting).

Table S2 Sn K-edge EXAFS fitting results for Ag/Sn-SnO_2, Sn-SnO_2, SnO_2, Sn foil, and SnO

sample	shell	N	σ^2 (Å^2)	ΔE_0 (eV)	R (Å)	R-factor
Sn foil	Sn-Sn1	4	10.5±1.0	0.4±0.8	3.00±0.00	0.020
	Sn-Sn2	8	4.9±1.4		3.74±0.07	
SnO_2	Sn-O	6.5±0.5	1.3±1.3	-1.0±0.9	2.15±0.01	0.017
	Sn-Sn1	1.5±0.5	0.1±2.0	-4.9±2.3	3.17±0.02	
	Sn-Sn2	4.4±1.8	0.1±2.0	4.3±2.1	3.70±0.02	
Sn-SnO_2	Sn-O	4.7±0.4	1.7±0.9	3.6±1.3	2.05±0.01	0.018
	Sn-Sn1	3.1±0.4		4.4±6.9	3.20±0.04	
	Sn-Sn2	4.0±0.9		3.3±2.1	3.72±0.01	
Ag/Sn-SnO_2	Sn-O	3.7±0.3	2.8±0.9	3.3±1.8	2.04±0.01	0.018
	Sn-Ag	2.0±0.7		5.8±2.5	3.01±0.02	
	Sn-Sn1	2.1±0.7		3.3±2.1	3.15±0.02	
	Sn-Sn2	3.2±0.9			3.70±0.01	

N: Coordination numbers; σ: Debye-Waller parameters, R: coordination distances; R-factor: goodness of fit, ΔE_0 energy shift. S_0^2 is amplitude reduction factor (S_0^2=0.88 was obtained by Sn foil fitting and applied for other sample fitting)

Table S3 Comparison of the catalytic performance of various electrocatalysts for CO_2RR to formate production in H-type reactor

Catalyst	Electrolyte	Potential (V *vs.* RHE)	$j_{formate}$ (mA cm^{-2})	$FE_{formate}$ (%)	Reference
**Ag/Sn-SnO_2**	**0.5 M $KHCO_3$**	**–1.37**	**48**	**90**	**This work**
InSn-3	0.1 M $KHCO_3$	–0.9	34.15	86	S1
Cu_6Sn_5/Sn	0.5 M $NaHCO_3$	–1.15	31	70	S2
CuSn-3	0.5 M $KHCO_3$	–1.0	26	90	S3
H-SnS_2	0.1 M $KHCO_3$	–1.0	25	87	S4
Ag-Sn/GO	0.5 M $NaHCO_3$	–0.94	21.3	88.3	S5
Cu@Sn nanocones	0.5 M $NaHCO_3$	–1.1	16	85	S6
Ag-Sn	0.5 M $NaHCO_3$	–0.8	16	80	S7
SnO_2QWs	0.1 M $KHCO_3$	–1.156	13.7	85	S8
SnO_xNPs	0.5 M $NaHCO_3$	–1.2	9	80	S9
SnO_x	0.1 M $KHCO_3$	–0.75	6.7	80	S10

Table S4 Comparison of the catalytic performances of various electrocatalysts for CO_2RR to formate production in Flow-type reactor

Catalyst	Electrolyte	Potential (V *vs.* RHE)	$j_{formate}$ (mA cm^{-2})	$FE_{formate}$ (%)	Stability	Reference
**Ag/Sn-SnO_2**	**1 M KOH**	**–2.0**	**2000**	**90**	**200 h; 200 mA cm^{-2}**	**This work**
Sn-ene QDs	1 M KOH	–2.0	923	89	100 h; 200 mA cm^{-2}	S11
InP QDs	3 M KOH	–2.6	930	90	4 h; 400 mA cm^{-2}	S12
Bi flakes	1 M KOH	–0.7	677	83	6 h; 500 mA cm^{-2}	S13
Bi-ene-NW	1 M KOH	–0.97	570	91	18 h; 200 mA cm^{-2}	S14
$ZnIn_2S_4$	1 M KOH	–1.0	490	93	60 h; 300 mA cm^{-2}	S15
Bi NSs	1 M KOH	–0.67	400	89	10 h; 197 mA cm^{-2}	S16
$Sn_{2.7}Cu$	1 M KOH	–0.7	370	84	40 h; 243 mA cm^{-2}	S17
Ni-In_2O_3@C	1 M KOH	–1.0	350	90	55 h; 200 mA cm^{-2}	S18
FTO/C	1 M $KHCO_3$	–1.0	330	90	160 h; 100 mA cm^{-2}	S19
Pits-Bi NS	1 M KOH	–1.5	325	92	110 h; 200 mA cm^{-2}	S20

Table S5 Comparison of the catalytic performances of various electrocatalysts for CO_2RR to pure HCOOH solution in the MEA with PSE layer reactor

Catalyst	j_{total} (mA)	j_{HCOOH} (mA cm^{-2})	FE_{HCOOH} (%)	Stability (h; mAcm^{-2}; moL L^{-1})	Reference
**Ag/Sn-SnO_2**	**1000**	**350**	**90**	**200 h; 100 mA cm^{-2}; 0.14[a]**	**This work**
	**800**	**270**	**90**	**25 h; 100 mA cm^{-2}; 0.27[b]**	
In_2O_3@C	320	80	\	3 h; 30 mA cm^{-2}; \[a]	S21
2D-Bi	800	200	78	100 h; 30 mA cm^{-2}; 0.1[a]	S22
Bi MS	220	175	84	110 h; 160 mA cm^{-2}; 0.13[a]	S23
nBuLi-Bi	800	400	88	100 h; 30 mA cm^{-2}; 0.1[a]	S24
	300	150	85	30 h; 30 mA cm^{-2}; 0.35[c]	
Pb_1Cu	900	225	90	180 h; 100 mA cm^{-2}; 0.1[a]	S25
BiSn	250	225	90	12 h; 100 mA cm^{-2}; 0.41[a]	S26

[a] Formic acid was released by feeding a flow of deionized water (anode-coupled OER reaction); [b, c] Formic acid was released by feeding a flow of N_2 vapour flow (b: anode-coupled OER reaction, c: anode-coupled HOR reaction)

Supplementary References

[S1] J. Wang, S. Ning, M. Luo, D. Xiang, W. Chen, X. Kang, Z. Jiang, S. Chen, In-Sn alloy core-shell nanoparticles: In-doped SnOx shell enables high stability and activity towards selective formate production from electrochemical reduction of CO2, Appl. Catal. B: Environ. 288 (2021) 119979. https://doi.org/10.1016/j.apcatb.2021.119979

[S2] Y. Wang, Y. Chen, Y. Zhao, J. Yu, Z. Liu, Y. Shi, H. Liu, X. Li, W. Zhou, Laser-fabricated channeled Cu6Sn5/Sn as electrocatalyst and gas diffusion electrode for efficient CO2 electroreduction to formate. Appl. Catal. B: Environ. 307 (2022) 120991. https://doi.org/10.1016/j.apcatb.2021.120991

[S3] M. Ren, H. Zheng, J. Lei, J. Zhang, X. Wang, B.I. Yakobson, Y. Yao, J.M. Tour, CO2 to formic acid using Cu-Sn on laser-induced graphene. ACS Appl. Mater. Interfaces 12 (2020) 41223-41229. https://doi.org/10.1021/acsami.0c08964

[S4] A. Zhang, Y. Liang, H. Li, S. Wang, Q. Chang, K. Peng, Z. Geng, J. Zeng, Electronic tuning of SnS2 nanosheets by hydrogen incorporation for efficient CO2 electroreduction. Nano Lett. 21 (2021) 7789-7795. https://doi.org/10.1021/acs.nanolett.1c02757

[S5] X. Zhang, F. Li, Y. Zhang, Alan M. Bond, J. Zhang, Stannate derived bimetallic nanoparticles for electrocatalytic CO2 reduction, J. Mater. Chem. A, 6 (2018) 7851-7858. https://doi.org/10.1039/C8TA02429D

[S6] C. Chen, Y. Pang, F. Zhang, J. Zhong, B. Zhang, Z. Cheng, Sharp Cu@ Sn nanocones on Cu foam for highly selective and efficient electrochemical reduction of CO2 to formate, J. Mater. Chem. A, 6 (2018) 19621-19630. https://doi.org/10.1039/C8TA06826G

[S7] W. Luc, C. Collins, S. Wang, H. Xin, K. He, Y. Kang, F. Jiao, Ag-Sn bimetallic catalyst with a core-shell structure for CO2 reduction. J. Am. Chem. Soc. 139 (2017) 1885-1893. https://doi.org/10.1021/jacs.6b10435

[S8] S. Liu, J. Xiao, X.F. Lu, J. Wang, X. Wang, X.W. Lou, Efficient electrochemical reduction of CO2 to HCOOH over sub-2 nm SnO2 quantum wires with exposed grain boundaries. Angew. Chem. Inter. Ed. 58 (2019) 8499-8503. https://doi.org/10.1002/anie.201903613

[S9] M.K. Kim, H. Lee, J.H. Won, W. Sim, S.J. Kang, H. Choi, M. Sharma, H.S. Oh, S. Ringe, Y. Kwon, H.M. Jeong, Design of less than 1 nm scale spaces on SnO2 nanoparticles for high-performance electrochemical CO2 reduction. Adv. Funct. Mater. 32 (2021) 2107349. https://doi.org/10.1002/adfm.202107349

[S10] Y. Cheng, J. Hou, P. Kang, Integrated capture and electroreduction of flue gas CO2 to formate using amine functionalized SnOx nanoparticles. ACS Energy Lett. 6 (2021) 3352-3358. https://doi.org/10.1021/acsenergylett.1c01553

[S11] M. Zhang, S. Zhou, W. Wei, D.-D. Ma, S.-G. Han, X. Li, X.-T. Wu, Q. Xu, Q.-L. Zhu, Few-atom-layer metallene quantum dots toward CO2 electroreduction at ampere-level current density and Zn-CO2 battery. Chem Catal. 2 (2022) 3528-3545. https://doi.org/10.1016/j.checat.2022.10.001

[S12] I. Grigioni, L.K. Sagar, Y.C. Li, G. Lee, Y. Yan, K. Bertens, R.K. Miao, X. Wang, J. Abed, D.H. Won, F.P. García de Arquer, A.H. Ip, D. Sinton, E.H. Sargent, CO2 electroreduction to formate at a partial current density of 930 mA cm^{-2} with InP colloidal quantum dot derived catalysts. ACS Energy Lett. 6 (2020) 79-84. https://doi.org/10.1021/acsenergylett.0c02165

[S13] Z. Xing, X. Hu, X. Feng, Tuning the microenvironment in gas-diffusion electrodes enables high-rate CO2 electrolysis to formate. ACS Energy Lett. 6 (2021) 1694-1702. https://doi.org/10.1021/acsenergylett.1c00612

[S14] M. Zhang, W. Wei, S. Zhou, D.-D. Ma, A. Cao, X.-T. Wu, Q.-L. Zhu, Engineering a conductive network of atomically thin bismuthene with rich defects enables CO2 reduction to formate with industry-compatible current densities and stability, Energy Environ. Sci. 14 (2021) 4998-5008. https://doi.org/10.1039/D1EE01495A

[S15] L.P. Chi, Z.Z. Niu, X.L. Zhang, P.P. Yang, J. Liao, F.Y. Gao, Z.Z. Wu, K.B. Tang, M.R. Gao, Stabilizing indium sulfide for CO2 electroreduction to formate at high rate by zinc incorporation. Nat. Commun. 12 (2021) 5835. https://doi.org/10.1038/s41467-021-26124-y

[S16] J. Yang, X. Wang, Y. Qu, X. Wang, H. Huo, Q. Fan, J. Wang, L. M. Yang, Y. Wu, Bi-based metal-organic framework derived leafy bismuth nanosheets for carbon dioxide electroreduction. Adv. Energy Mater. 10 (2020) 2001709. https://doi.org/10.1002/aenm.202001709

[S17] K. Ye, Z. Zhou, J. Shao, L. Lin, D. Gao, N. Ta, R. Si, G. Wang, X.J.A.C.I.E. Bao, In situ reconstruction of a hierarchical Sn-Cu/SnOx core/shell catalyst for high-performance CO2 electroreduction. Angew. Chem. Int. Ed. 59 (2020) 4814-4821. https://doi.org/10.1002/anie.201916538

[S18] Z. Chen, G. Yu, B. Li, X. Zhang, M. Jiao, N. Wang, X. Zhang, L. Liu, In situ carbon encapsulation confined nickel-doped indium oxide nanocrystals for boosting CO2 electroreduction to the industrial level. ACS Catal. 11 (2021) 14596-14604. https://doi.org/10.1021/acscatal.1c04182

[S19] Y.J. Ko, J.Y. Kim, W.H. Lee, M.G. Kim, T.Y. Seong, J. Park, Y. Jeong, B.K. Min, W.S. Lee, D.K. Lee, H.S. Oh, Exploring dopant effects in stannic oxide nanoparticles

for CO2 electro-reduction to formate. Nat. Commun. 13 (2022) 2205. https://doi.org/10.1038/s41467-022-29783-7

[S20] Y. Yuan, Q. Wang, Y. Qiao, X. Chen, Z. Yang, W. Lai, T. Chen, G. Zhang, H. Duan, M. Liu, H. Huang, In situ structural reconstruction to generate the active sites for CO2 electroreduction on bismuth ultrathin nanosheets. Adv. Energy Mater. 12 (2022) 2200970. https://doi.org/10.1002/aenm.202200970

[S21] Z. Wang, Y. Zhou, D. Liu, R. Qi, C. Xia, M. Li, B. You, B.Y. Xia, Carbon-confined indium oxides for efficient carbon dioxide reduction in a solid-state electrolyte flow cell. Angew. Chem. Inter. Ed. 61 (2022) e202200552. https://doi.org/10.1002/anie.202200552

[S22] C. Xia, P. Zhu, Q. Jiang, Y. Pan, W. Liang, E. Stavitski, H.N. Alshareef, H. Wang, Continuous production of pure liquid fuel solutions via electrocatalytic CO2 reduction using solid-electrolyte devices. Nat. Energy 4 (2019) 776-785. https://doi.org/10.1038/s41560-019-0451-x

[S23] Y. Zhang, R. Zhang, F. Chen, F. Zhang, Y. Liu, X. Hao, H. Jin, X. Zhang, Z. Lu, H. Dong, F. Lu, W. Wang, H. Liu, Y. Chen, Mass-transfer-enhanced hydrophobic Bi microsheets for highly efficient electroreduction of CO2 to pure formate in a wide potential window. Appl Catal B: Environ 322 (2023) 122127. https://doi.org/10.1016/j.apcatb.2022.122127

[S24] L. Fan, C. Xia, P. Zhu, Y. Lu, H. Wang, Electrochemical CO2 reduction to high-concentration pure formic acid solutions in an all-solid-state reactor. Nat. Commun. 11 (2020) 3633. https://doi.org/10.1038/s41467-020-17403-1

[S25] T. Zheng, C. Liu, C. Guo, M. Zhang, X. Li, Q. Jiang, W. Xue, H. Li, A. Li, C.-W. Pao, J. Xiao, C. Xia, J. Zeng, Copper-catalysed exclusive CO2 to pure formic acid conversion via single-atom alloying. Nat. Nanotech. 16 (2021) 1386-1393. https://doi.org/10.1038/s41565-021-00974-5

[S26] L. Li, Z. Liu, X. Yu, M. Zhong, Achieving high single-pass carbon conversion efficiencies in durable CO2 electroreduction in strong acids via electrode structure engineering. Angew. Chem. Inter. Ed. 15 (2023) e202300226. https://doi.org/10.1002/anie.202300226
